# Daratumumab, lenalidomide, and dexamethasone in transplant-ineligible newly diagnosed multiple myeloma: MAIA final survival analysis

**DOI:** 10.1038/s41375-026-03097-9

**Published:** 2026-08-24

**Authors:** Thierry Facon, Shaji K. Kumar, Robert Z. Orlowski, Nizar Bahlis, Philippe Moreau, Hartmut Goldschmidt, Supratik Basu, Cyrille Hulin, Sonja Zweegman, Katja Weisel, Aurore Perrot, Caroline Jacquet, Noopur Raje, Salomon Manier, Ajai Chari, Markus Hansson, Mohamad Mohty, Michel Delforge, Torben Plesner, Gordon Cook, Xavier Leleu, Hang Quach, Christopher P. Venner, Mourad Tiab, Margaret Macro, Laurent Frenzel, George Wang, Huiling Pei, Kasey Bolyard, Robin Carson, Fredrik Borgsten, Saad Z. Usmani

**Affiliations:** 1 University of Lille, CHU de Lille, Service des Maladies du Sang, Lille, France; 2 Department of Hematology, Mayo Clinic Rochester, Rochester, MN USA; 3 Department of Lymphoma & Myeloma, The University of Texas MD Anderson Cancer Center, Houston, TX USA; 4 Arnie Charbonneau Cancer Research Institute, University of Calgary, Calgary, AB Canada; 5 Hematology Department, University Hospital Hôtel-Dieu, Nantes, France; 6 Internal Medicine V, Hematology, Oncology and Rheumatology, GMMG Study Group, Heidelberg University Hospital and National Center for Tumor Diseases, Heidelberg, Germany; 7 Royal Wolverhampton NHS Trust and University of Wolverhampton, CRN West Midlands, NIHR, Wolverhampton, UK; 8 Department of Hematology, Hôpital Haut Lévêque, University Hospital, Pessac, France; 9 Department of Hematology, Amsterdam University Medical Center, Vrije Universiteit Amsterdam, Cancer Center Amsterdam, Amsterdam, The Netherlands; 10 Department of Oncology, Hematology and Bone Marrow Transplantation with Section of Pneumology, University Medical Center Hamburg-Eppendorf, Hamburg, Germany; 11 Université de Toulouse, Centre Hospitalo-Universitaire, IUCT-Oncopole, Service d’Hématologie, Toulouse, France; 12 Hematology Department, University Hospital, Nancy, France; 13 Center for Multiple Myeloma, Massachusetts General Hospital Cancer Center, Boston, MA USA; 14 University of Lille, CHU de Lille, Hôpital Huriez, Service d’Hématologie, Lille, France; 15 Department of Medicine, Hematology and Medical Oncology, Icahn School of Medicine at Mount Sinai, New York, NY USA; 16 Department of Hematology, Skåne University Hospital, Lund and Sahlgrenska University Hospital, Göteborg, Sweden; 17 Department of Clinical Hematology and Cellular Therapy, Saint-Antoine Hospital, Sorbonne University, Paris, France; 18 University Hospital Leuven, Leuven, Belgium; 19 Vejle Hospital and University of Southern Denmark, Vejle, Denmark; 20 NIHR Leeds BRC & CRUK Clinical Trials Unit, University of Leeds, Leeds, UK; 21 CHU de Poitiers, Hôpital la Milétrie, Poitiers, France; 22 University of Melbourne, St Vincent’s Hospital, Melbourne, VIC Australia; 23 Department of Medical Oncology, Cross Cancer Institute, University of Alberta, Edmonton, AB Canada; 24 BC Cancer – Vancouver Centre, University of British Columbia, Vancouver, BC Canada; 25 CHD Vendée, La Roche sur Yon, France; 26 CHU de Caen, Caen, France; 27 Hôpital Necker-Enfants Malades, Paris, France; 28 Johnson & Johnson, Spring House, PA USA; 29 Johnson & Johnson, Titusville, NJ USA; 30 Johnson & Johnson, Raritan, NJ USA; 31 Memorial Sloan Kettering Cancer Center, New York, NY USA

**Keywords:** Targeted therapies, Myeloma

## Abstract

In previous analyses of MAIA, daratumumab plus lenalidomide/dexamethasone (D-Rd) significantly improved progression-free survival and overall survival (OS) versus lenalidomide/dexamethasone (Rd) in transplant-ineligible newly diagnosed multiple myeloma (NDMM). We report results on long-term OS and subsequent antimyeloma therapies from the MAIA final analysis. A protocol amendment (July 20, 2021) led to a long-term extension of MAIA, during which patients were followed for OS. A total of 737 patients were randomized to D-Rd (*n* = 368) or Rd (*n* = 369). At a median follow-up of 89.3 months (range, 0.0–102.2; interquartile range, 85.3–93.0), median OS was 90.3 months (95% confidence interval [CI], 80.8–not estimable [NE]) with D-Rd versus 64.1 months (56.0–70.8) with Rd (hazard ratio [HR], 0.67; 95% CI, 0.55–0.82); estimated 7-year OS rates were 53.1% (95% CI, 47.8–58.2) and 39.3% (34.1–44.5), respectively. Median time to subsequent antimyeloma treatment was not reached (95% CI, 84.1–NE) for D-Rd versus 42.4 months (33.5–50.6) for Rd (HR, 0.51; 95% CI, 0.41–0.63; *P* < 0.0001). Death due to adverse events occurred in 84 patients (D-Rd, *n* = 44/364 [12%]; Rd, *n* = 40/365 [11%]). With >7 years of follow-up, D-Rd demonstrated a new benchmark for median OS (7.5 years) in transplant-ineligible NDMM, further supporting frontline D-Rd use to maximize survival.

## Introduction

Daratumumab is a human IgGκ monoclonal antibody targeting CD38 with direct on-tumor [1–4] and immunomodulatory [5–7] mechanisms of action. The inclusion of daratumumab in frontline combination therapy has consistently demonstrated clinical efficacy in patients with newly diagnosed multiple myeloma (NDMM) [8–16].

MAIA is a phase 3 randomized study that evaluated the combination of daratumumab plus lenalidomide/dexamethasone (D-Rd) compared with lenalidomide/dexamethasone (Rd) alone in transplant-ineligible patients with NDMM. At the first prespecified interim analysis of MAIA, at a median follow-up of 28.0 months, D-Rd significantly improved progression-free survival (PFS) versus Rd (hazard ratio [HR], 0.56; 95% confidence interval [CI], 0.43–0.73; *P* < 0.001) and achieved deeper responses, including higher rates of complete response or better (≥CR; 47.6% vs 24.9%; *P* < 0.001) and minimal residual disease (MRD) negativity (10^–5^; 24.2% vs 7.3%; *P* < 0.001) [12]. After a median follow-up of 56.2 months, D-Rd significantly improved overall survival (OS) versus Rd (HR, 0.68; 95% CI, 0.53–0.86; *P* = 0.0013), and no new safety concerns were observed [13]. With longer follow-up, at a median of 64.5 months, the addition of daratumumab to Rd continued to demonstrate deep and durable responses, including sustained MRD negativity lasting for ≥12 months at the 10^–5^ sensitivity threshold (18.8% [D-Rd] vs 4.1% [Rd]; *P* < 0.0001), translating to PFS and OS benefits in transplant-ineligible patients with NDMM [17].

Herein, we report the findings from a long-term follow-up analysis of OS from MAIA, including results from a post hoc subgroup analysis of OS based on age (<70 years, ≥70 to <75 years, ≥75 years, and ≥80 years), as well as updated results on subsequent antimyeloma therapies and death-related safety results, after a median follow-up of approximately 7.5 years.

## Methods

### Study design and participants

MAIA (ClinicalTrials.gov Identifier: NCT02252172) was a randomized, open-label, active-controlled, parallel-group, multicenter, phase 3 study. The study design and complete eligibility criteria have been published previously [12]. Briefly, patients included in the study were aged ≥18 years, had documented NDMM [18], had an Eastern Cooperative Oncology Group performance status score of 0 to 2, and were not candidates for high-dose chemotherapy and autologous stem cell transplant because of age (≥65 years) or the presence of comorbidities likely to have a negative impact on the tolerability of high-dose chemotherapy with autologous stem cell transplant.

### Ethics approval and consent to participate

The study protocol was approved by the independent ethics committee or institutional review board at each site (Supplementary Table 1), and all patients provided written informed consent. All methods were performed in accordance with the relevant guidelines and regulations. The trial was conducted in accordance with the principles of the Declaration of Helsinki and the International Conference on Harmonisation Good Clinical Practice guidelines.

### Randomization and study treatment

Patients were randomly assigned 1:1 to receive treatment with D-Rd or Rd and were stratified by International Staging System (ISS) disease stage (I vs II vs III), geographic region (North America vs other), and age (<75 years vs ≥75 years). All patients received 28-day cycles of lenalidomide (25 mg orally once daily on Days 1–21 of each 28-day cycle; 10 mg was recommended for patients with creatinine clearance 30–50 mL/min) and dexamethasone (40 mg orally on Days 1, 8, 15, and 22; 20 mg was recommended if patients were aged >75 years or had a body mass index <18.5 kg/m^2^) until disease progression or unacceptable toxicity. Patients in the D-Rd arm received daratumumab (16 mg/kg intravenously) once weekly during Cycles 1 and 2, once every 2 weeks during Cycles 3 through 6, and once every 4 weeks thereafter. At the discretion of the investigator, patients with no known allergies/intolerances to sorbitol who were initially randomized to D-Rd were given the option to switch from intravenous to subcutaneous administration of daratumumab on Day 1 of any cycle, per the amended protocol. This amendment was made to provide flexibility to prioritize patient safety during the COVID-19 pandemic. Treatment was stopped if disease progression or unacceptable adverse events occurred. During the follow-up phase, which began once a patient discontinued all study treatments, patients were evaluated for subsequent antimyeloma therapy, PFS on the next line of therapy (per investigator assessment), second primary malignancies, and survival. Patients remained in the follow-up phase until death, loss to follow-up, consent withdrawal, or study completion (whichever occurred first).

### Outcomes

The primary endpoint was PFS and has been previously reported [12]. Secondary endpoints included ≥CR rate, very good partial response or better (≥VGPR) rate, MRD-negativity rate, overall response rate, OS, and time to subsequent antimyeloma treatment. Durability of MRD negativity was also a prespecified exploratory endpoint. Secondary endpoints reported here include OS (defined as the date of randomization to the date of death due to any cause) and time to subsequent antimyeloma treatment (defined as the time from randomization to the start of subsequent antimyeloma treatment; death due to progressive disease without start of subsequent therapy was considered as an event). All efficacy endpoints are defined in the Supplementary Information.

Response and disease progression were assessed using a validated computer algorithm based on International Myeloma Working Group criteria [19, 20]. MRD assessments were performed by a central laboratory at baseline; at the time of suspected CR or stringent CR; and at 12, 18, 24, 30, 36, 48, and 60 months after Cycle 1 Day 1 (±1 month) for patients with a near CR or better. MRD was evaluated via next-generation sequencing using the clonoSEQ assay (version 2.0; Adaptive Biotechnologies, Seattle, WA, USA). Cytogenetic risk was assessed locally by fluorescence in situ hybridization or karyotype testing; high cytogenetic risk was defined as the presence of ≥1 of the following cytogenetic abnormalities: del(17p), t(14;16), or t(4;14).

On July 20, 2021, a protocol amendment led to a long-term extension of MAIA, during which patients were followed for OS. Data collection in the clinical database was limited to subsequent antimyeloma therapies, survival follow-up, death-related information, end-of-treatment disposition, and end-of-study disposition. For this long-term analysis, data on adverse events were not captured, per protocol; safety data captured were limited to death-related data.

### Statistical analysis

Complete statistical methods have been previously published [12, 13]. The primary analysis was performed in the intention-to-treat (ITT) population, which included all patients who underwent randomization. The safety population included all patients who received ≥1 dose of study treatment. After reaching statistical significance at the second interim OS analysis, the long-term survival follow-up was extended to approximately 390 deaths, when the median survival for the D-Rd group would likely be reached. Time-to-event endpoints, including OS and time to subsequent antimyeloma treatment, were compared between the D-Rd and Rd treatment groups using a stratified log-rank test with randomization stratification factors. The Kaplan-Meier method was used to estimate distributions. For the entire ITT population, HRs and 95% CIs were calculated using a stratified Cox regression model (stratification factors: ISS disease stage [I vs II vs III], region [North America vs other], and age [<75 years vs ≥75 years]) with treatment as the sole explanatory variable. For patient subgroups within the ITT population, HRs and 95% CIs were calculated using a nonstratified Cox regression model with treatment as the sole variable. Post hoc subgroup analyses of OS by patient age (<70 years, ≥70 to <75 years, ≥75 years, and ≥80 years) and OS by MRD status and sustained MRD-negativity status were conducted and are reported here. All subgroup analyses reported herein were exploratory in nature. Descriptive statistics were used to summarize data, including information on classes of subsequent therapies, subsequent regimens, rate of study treatment discontinuation, and causes of death. SAS (version 9.4) was used for all statistical analyses.

### Role of the funding source

The study sponsor funded the study and, in collaboration with the authors, designed the trial and collected, analyzed, and interpreted the data. All authors had full access to all of the data in the study and had responsibility for the decision to submit for publication.

## Results

**Fig. 1:**
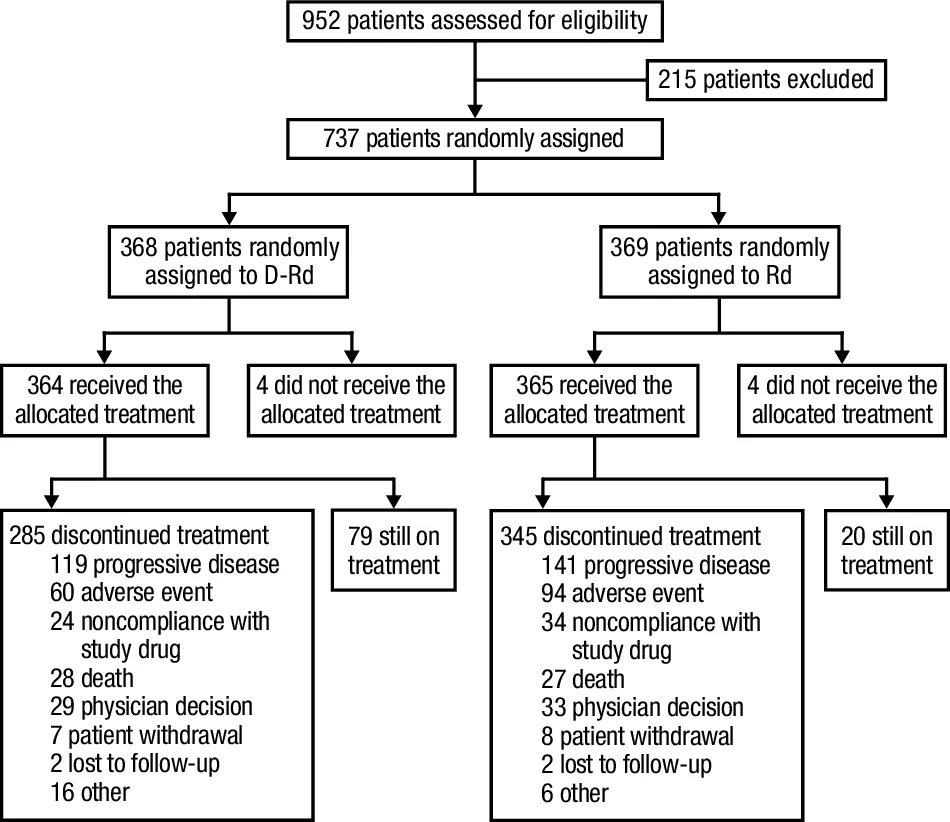
Trial profile. D-Rd daratumumab plus lenalidomide/dexamethasone, Rd lenalidomide/dexamethasone.

A total of 737 patients were randomized in the MAIA study to D-Rd (*n* = 368) or Rd (*n* = 369; Fig. 1). Baseline patient characteristics for the ITT population and for subgroups based on patient age were previously published [12] and were well balanced between treatment groups (Supplementary Table 2) [17]. The median patient age was 73 years (range, 45–90; interquartile range [IQR], 70–78). In the D-Rd and Rd groups, 78/368 (21%) patients and 77/369 (21%) patients, respectively, were aged <70 years, 130 (35%) patients and 131 (36%) patients were aged ≥70 to <75 years, and 160 (43%) patients and 161 (44%) patients were aged ≥75 years (with 66 [18%] patients and 71 [19%] patients aged ≥80 years). Based on the simplified frailty score criteria [21, 22], 172/368 (47%) patients in the D-Rd group and 169/369 (46%) patients in the Rd group were classified as frail [23]. Most patients who were randomized received ≥1 dose of study treatment (D-Rd, 364/368 [99%]; Rd, 365/369 [99%]; Fig. 1). At the time of the clinical data cutoff for this final OS analysis (November 30, 2023), 285/364 (78%) patients in the D-Rd group and 345/365 (95%) patients in the Rd group had discontinued treatment. The most common reasons for treatment discontinuation were progressive disease (D-Rd, 119/364 [33%]; Rd, 141/365 [39%]) and adverse events (60/364 [16%] vs 94/365 [26%]).

**Fig. 2:**
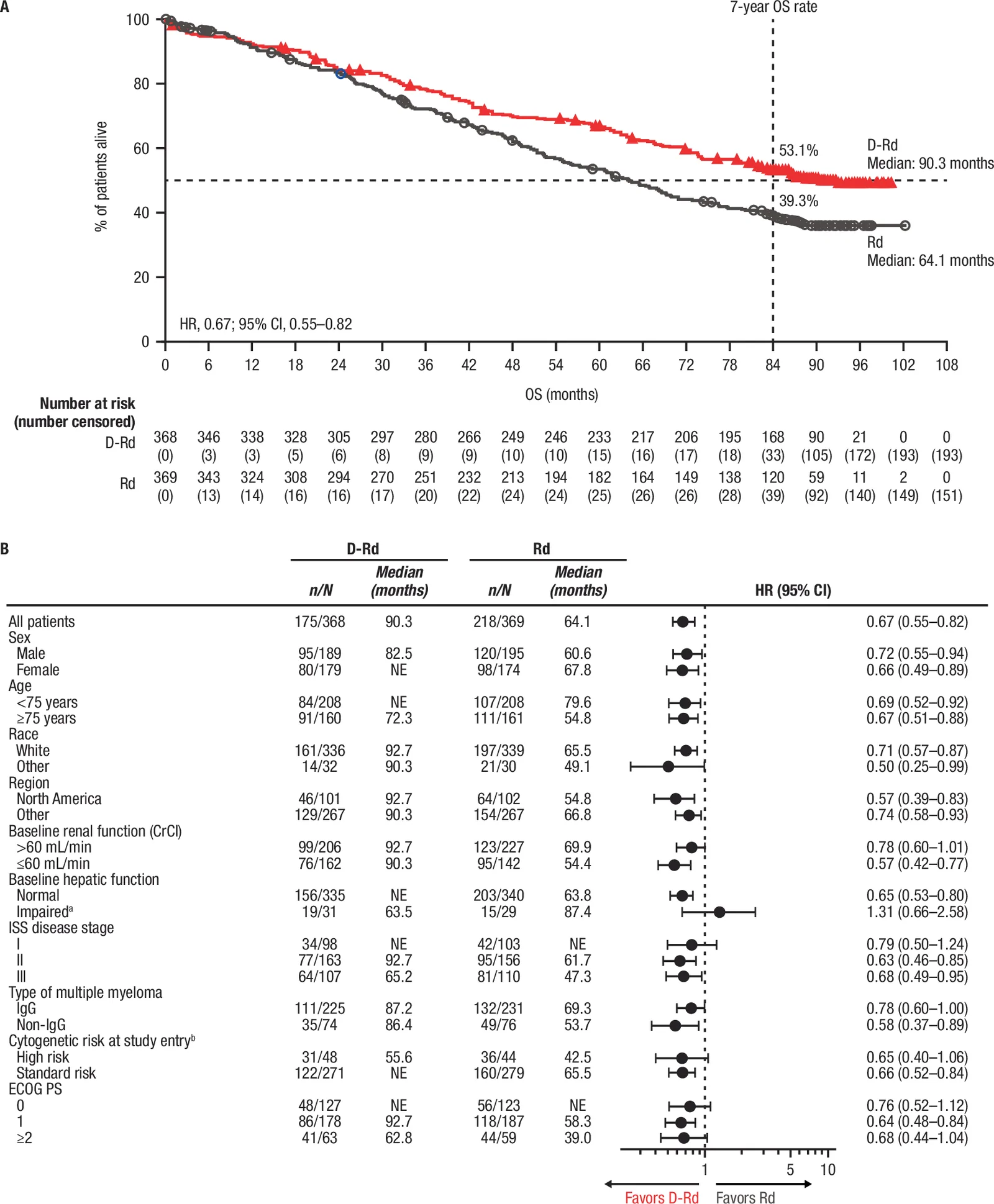
OS in the ITT population. **A** Kaplan-Meier estimates of OS in the ITT population. **B** Exploratory analysis of OS in subgroups of the ITT population that were defined according to baseline characteristics. For each subgroup parameter, the interaction term was tested for OS through a Cox model with variables of treatment group, subgroup covariate, and interaction of treatment by subgroup covariate. The *P*-values for the interaction terms are as follows: sex (*P* = 0.73), age (*P* = 0.92), race (*P* = 0.35), region (*P* = 0.30), baseline renal function (*P* = 0.11), baseline hepatic function (*P* = 0.041), ISS disease stage (*P* = 0.55 and *P* = 0.74 for ISS stage I and II, respectively), type of multiple myeloma (*P* = 0.24), cytogenetic risk at study entry (*P* = 0.97), and ECOG PS (*P* = 0.64 and *P* = 0.82 for ECOG PS score of 0 and 1, respectively). OS overall survival, ITT intention-to-treat, D-Rd daratumumab plus lenalidomide/dexamethasone, Rd lenalidomide/dexamethasone, HR hazard ratio, CI confidence interval, NE not estimable, CrCl creatinine clearance, ISS International Staging System, ECOG PS Eastern Cooperative Oncology Group performance status, ULN upper limit of normal, AST aspartate aminotransferase. ^a^Impaired hepatic function included mild (total bilirubin ≤ULN and AST > ULN, or total bilirubin >ULN but ≤1.5×ULN), moderate (total bilirubin >1.5×ULN but ≤3×ULN), and severe (total bilirubin >3×ULN) impairment. ^b^Patients with high cytogenetic risk were positive by fluorescence in situ hybridization or karyotype testing for ≥1 of the following cytogenetic abnormalities: del(17p), t(14;16), or t(4;14).

**Fig. 3:**
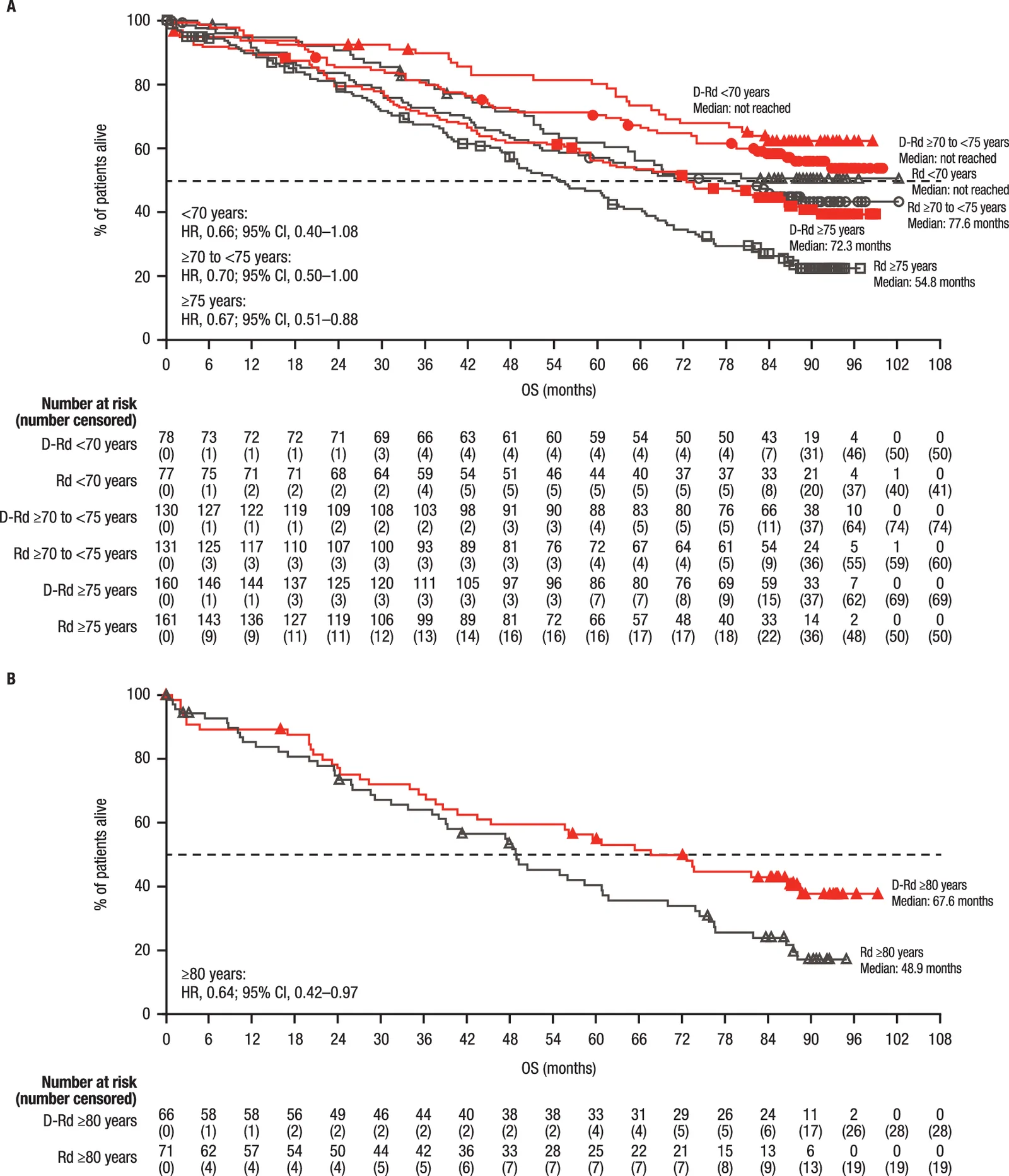
OS by age subgroup in the ITT population. Kaplan-Meier estimates of **A** OS in patients aged <70 years, ≥70 to <75 years, and ≥75 years and **B** OS in patients aged ≥80 years. OS overall survival, ITT intention-to-treat, D-Rd daratumumab plus lenalidomide/dexamethasone, Rd lenalidomide/dexamethasone, HR hazard ratio, CI confidence interval.

At a median follow-up of 89.3 months (range, 0.0–102.2; IQR, 85.3–93.0) in the ITT population overall (89.6 months [range, 0.0–99.9; IQR, 85.2–93.4] in the D-Rd group and 88.9 months [range, 0.0–102.2; IQR, 85.5–92.3] in the Rd group), 175 (48%) patients in the D-Rd group and 218 (59%) patients in the Rd group had died. Median OS was 90.3 months (95% CI, 80.8–not estimable [NE]) with D-Rd versus 64.1 months (95% CI, 56.0–70.8) with Rd (HR, 0.67; 95% CI, 0.55–0.82; Fig. 2A). Estimated 7-year OS rates were 53.1% (95% CI, 47.8–58.2) in the D-Rd group and 39.3% (95% CI, 34.1–44.5) in the Rd group. Prespecified subgroup analyses of OS based on baseline characteristics are shown in Fig. 2B. Subgroup analyses of OS trended in favor of D-Rd versus Rd across subgroups, including patients aged <70 years (median not reached [95% CI, NE–NE] vs not reached [57.2–NE]; HR, 0.66; 95% CI, 0.40–1.08), ≥70 to <75 years (median not reached [82.1–NE] vs 77.6 months [55.1–NE]; HR, 0.70; 95% CI, 0.50–1.00), ≥75 years (median 72.3 months [58.8–86.7] vs 54.8 months [47.3–63.1]; HR, 0.67; 95% CI, 0.51–0.88), and ≥80 years (median 67.6 months [40.6–NE] vs 48.9 months [37.1–60.9]; HR, 0.64; 95% CI, 0.42–0.97; Fig. 3).

Given that no further MRD data were collected since the time of the previous analysis of MAIA (median follow-up 64.5 months), rates of MRD negativity and sustained MRD negativity for ≥12 months remained unchanged since the previous analysis [17]. As previously reported, rates of MRD negativity and sustained MRD negativity for ≥12 months at the 10^–5^ sensitivity threshold were higher in the D-Rd group versus the Rd group (MRD-negativity rates: 118/368 [32%] vs 41/369 [11%]; *P* < 0.0001; sustained MRD-negativity rates: 69 [19%] vs 15 [4%]; *P* < 0.0001) [17]. When OS was assessed by MRD and sustained MRD status, patients who achieved MRD negativity and sustained MRD negativity had prolonged OS versus patients who did not (Supplementary Figs. 1 and 2). However, D-Rd prolonged OS versus Rd, regardless of MRD status and in patients who did not achieve sustained MRD negativity.

**Fig. 4:**
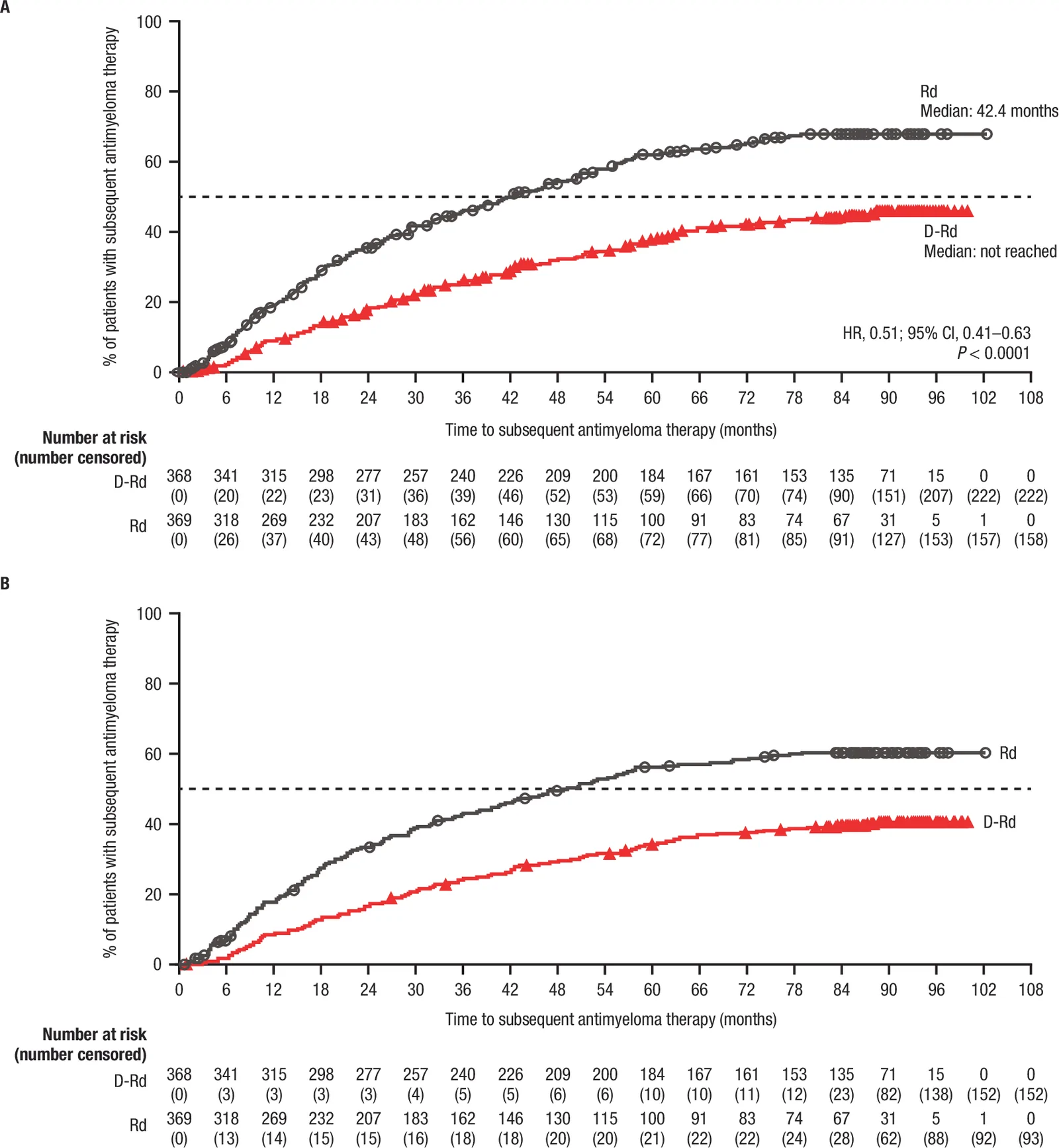
Time to subsequent antimyeloma treatment in the ITT population. **A** Kaplan-Meier estimates of time to subsequent antimyeloma treatment. **B** Post hoc competing risks analysis of time to subsequent antimyeloma treatment. Patient deaths due to nonprogression reasons were considered as competing events. ITT intention-to-treat, Rd lenalidomide/dexamethasone, D-Rd daratumumab plus lenalidomide/dexamethasone, HR hazard ratio, CI confidence interval.

Within the ITT population, 146 (40%) of 368 patients in the D-Rd group and 211 (57%) of 369 patients in the Rd group had an event of initiating subsequent antimyeloma therapy or death due to progressive disease (without receiving subsequent therapy). Median time to subsequent antimyeloma treatment was not reached (95% CI, 84.1–NE) in the D-Rd group versus 42.4 months (95% CI, 33.5–50.6) in the Rd group (HR, 0.51; 95% CI, 0.41–0.63; *P* < 0.0001; Fig. 4A). A consistent trend was observed in a post hoc analysis of time to subsequent antimyeloma treatment that considered deaths due to reasons other than disease progression as competing events (Fig. 4B). Among treated patients, 140 (38%) of 364 patients in the D-Rd group and 201 (55%) of 365 patients in the Rd group received ≥1 subsequent line of antimyeloma therapy on study. Across subsequent therapy lines, the most common antineoplastic agents after D-Rd and Rd, respectively, were bortezomib (28% vs 42%), daratumumab (6% vs 29%), and carfilzomib (7% vs 12%) in treated patients. No patient in either group reported the use of BCMA- or GPRC5D-targeted therapy. Two patients in the D-Rd group and two patients in the Rd group received investigational drugs in subsequent therapy lines.

Of the 341 total patients receiving subsequent antimyeloma therapy, proteasome inhibitor–based therapy was the most common first subsequent therapy class in both the D-Rd and Rd groups (69 [49%] of 140 patients vs 101 [50%] of 201 patients, respectively; Table 1). Daratumumab-containing first subsequent therapy regimens were received by 15 (11%) patients in the D-Rd group and 49 (24%) patients in the Rd group. Among patients who received and were evaluable for their best response to first subsequent antimyeloma therapy (D-Rd, *n* = 130; Rd, *n* = 193), ≥CR was achieved in 6 (5%) patients in the D-Rd group and 8 (4%) patients in the Rd group; ≥VGPR was achieved in 18 (14%) and 46 (24%) patients, respectively (Supplementary Fig. 3).

**Table 1:** Summary of first-line subsequent antimyeloma therapy in the safety population.

	D-Rd (*n* = 364)	Rd (*n* = 365)
Total number of patients with first-line subsequent therapy	140	201
First subsequent therapy class^a^		
Proteasome inhibitor only	69 (49)	101 (50)
Immunomodulatory drug only	22 (16)	25 (12)
Proteasome inhibitor + immunomodulatory drug	25 (18)	16 (8)
Daratumumab monotherapy or combination	15 (11)	49 (24)
Daratumumab monotherapy	1 (1)	2 (1)
﻿Other	9 (6)	10 (5)
Most common first subsequent therapy regimens^b^		
Bortezomib/cyclophosphamide/dexamethasone	19 (14)	29 (14)
Bortezomib/dexamethasone	20 (14)	28 (14)
Bortezomib/melphalan/prednisone	14 (10)	28 (14)
Daratumumab/bortezomib/dexamethasone	4 (3)	27 (13)
Lenalidomide/dexamethasone	13 (9)	16 (8)
Bortezomib/pomalidomide/dexamethasone	9 (6)	3 (1)
Bortezomib/lenalidomide/dexamethasone	8 (6)	3 (1)
Daratumumab/lenalidomide/dexamethasone	4 (3)	6 (3)
Pomalidomide/dexamethasone	2 (1)	6 (3)

Data are *n* (%). Percentages were calculated with the number of patients who received subsequent therapy in each treatment group as the denominator.

D-Rd daratumumab plus lenalidomide/dexamethasone, Rd lenalidomide/dexamethasone.

^a^Therapy classes are mutually exclusive. Patients in any therapy class subgroup may have received additional agents (other than proteasome inhibitor, immunomodulatory drug, or daratumumab), such as dexamethasone.

^b^Regimens received by ≥3% of patients in either treatment group.

Among the 228 patients in the D-Rd group and 168 patients in the Rd group who had not received subsequent antimyeloma therapy by the end of data collection, all had discontinued the study, with the most common reasons for discontinuation being death after study treatment (46 [20%] patients in the D-Rd group and 42 [25%] patients in the Rd group) and end of data collection (137 [60%] patients and 68 [40%] patients, respectively); death while on study treatment was reported as the reason for discontinuation for 30 (13%) patients in the D-Rd group and 34 (20%) patients in the Rd group (Supplementary Table 3). Notably, 79 (35%) patients and 20 (12%) patients who had not received subsequent antimyeloma therapy were still receiving D-Rd and Rd as frontline therapy, respectively, at the end of data collection.

In the safety population, deaths were reported for 173 (48%) of 364 patients in the D-Rd group and 218 (60%) of 365 patients in the Rd group, most frequently due to disease progression (D-Rd, 76 [21%]; Rd, 88 [24%]; Table 2). An adverse event was the primary cause of death for 44 (12%) patients in the D-Rd group and 40 (11%) patients in the Rd group; most of these events were considered unrelated to study treatment. Aside from disease progression and adverse events, primary causes of death occurring in ≥1% of patients in either treatment group were infections/infestations (2% in the D-Rd group and 8% in the Rd group), general disorders/administration-site conditions (related to the general health condition of the patient; 3% and 1%), neoplasms (benign, malignant, or unspecified; 3% and 1%), cardiac disorders (<1% and 2%), nervous system disorders (1% and 1%), and unknown (4% and 7%). Deaths within 30 days of the last study treatment dose occurred in 31 (9%) patients in the D-Rd group and 35 (10%) patients in the Rd group, most often due to adverse events (D-Rd, 29 [8%]; Rd, 32 [9%]).

**Table 2:** Summary of death and causes of death in the safety population.

	D-Rd (*n* = 364)	Rd (*n* = 365)
Total number of patients who died during the study	173 (48)	218 (60)
Primary cause of death		
Disease progression	76 (21)	88 (24)
Adverse event	44 (12)	40 (11)^a^
Related to study treatment^b^	14 (4)	10 (3)
Unrelated to study treatment	28 (8)	29 (8)
COVID-19	2 (1)	0
Other^c^	53 (15)	90 (25)
Infections/infestations	9 (2)	30 (8)
General disorders/administration-site conditions^d^	11 (3)	5 (1)
Neoplasms (benign, malignant, or unspecified)	11 (3)	4 (1)
Cardiac disorders	1 (<1)	8 (2)
Nervous system disorders	3 (1)	5 (1)
Unknown	13 (4)	27 (7)
Deaths within 30 days of last study treatment dose	31 (9)	35 (10)
Primary cause of death		
Disease progression	1 (<1)	1 (<1)
Adverse event	29 (8)	32 (9)
Related to study treatment^b^	11 (3)	10 (3)
Unrelated to study treatment	18 (5)	22 (6)
Other^e^	1 (<1)	2 (1)

Data are *n* (%).

D-Rd daratumumab plus lenalidomide/dexamethasone, Rd lenalidomide/dexamethasone.

^a^One patient in the Rd group had an adverse event as the primary cause of death, but it was not specified by the investigator whether the event was considered related or unrelated to study treatment.

^b^Adverse events were related to ≥1 of the three components of study treatment: daratumumab, lenalidomide, and dexamethasone.

^c^Other reasons were reported in ≥1% of patients in either treatment group.

^d^All events were related to the general health condition of the patient.

^e^Includes a nervous system disorder in one patient in the D-Rd group and a blood and lymphatic system disorder and general disorder/administration-site condition in one patient each in the Rd group.

## Discussion

In this final analysis of the MAIA study, median OS was reached for the first time in the D-Rd group after a median follow-up of approximately 7.5 years, and D-Rd continued to demonstrate a clinical survival benefit versus Rd alone in transplant-ineligible patients with NDMM. Median OS was 90.3 months (7.5 years) with D-Rd versus 64.1 months (5.3 years) with Rd, setting a new benchmark for median OS in the transplant-ineligible population. When OS was assessed based on age (<70 years, ≥70 to <75 years, ≥75 years, and ≥80 years), OS trended in favor of D-Rd versus Rd across age subgroups. The OS benefit with D-Rd versus Rd was generally consistent across prespecified patient subgroups, including patients with high cytogenetic risk. Rates of MRD negativity and sustained MRD negativity for ≥12 months, which remained unchanged since the previous analysis of MAIA (median follow-up 64.5 months) [17] due to lack of further MRD data collection, were higher with D-Rd versus Rd alone. When OS was assessed based on MRD and sustained MRD status, D-Rd prolonged OS versus Rd, regardless of MRD status and in patients who did not achieve sustained MRD negativity.

The proportion of patients who received ≥1 subsequent antimyeloma therapy was lower in the D-Rd group (38%) compared with the Rd group (55%), and median time to subsequent antimyeloma treatment was longer for D-Rd compared with Rd. These results suggest that patients in the D-Rd group experienced a longer duration of therapeutic benefit compared with patients in the Rd group. A total of 29% of patients treated with Rd alone (compared with only 6% of patients treated with D-Rd) received daratumumab-based regimens as subsequent antimyeloma therapy across therapy lines, with 24% of these patients in the Rd group (compared with only 11% in the D-Rd group) receiving daratumumab-containing regimens as their first subsequent therapy. It is notable that various regimens were approved/reimbursed in different phases in different countries throughout the study, which impacted choice of subsequent therapy in both treatment groups. In the MAIA study and other global clinical studies, these differences in the choice of subsequent therapy based on regimen approvals/reimbursements may have impacted OS. Among patients who had not received subsequent antimyeloma therapy by the end of data collection, a substantial proportion were still receiving D-Rd and Rd treatment (35% and 12%, respectively) at the time the study ended. Had these patients continued to be followed, additional data may have been captured indicating that some had received subsequent antimyeloma therapy, including subsequent daratumumab-containing therapy. No patient in either treatment group received BCMA- or GPRC5D-targeted therapy, and only two patients in the D-Rd group and two patients in the Rd group received investigational drugs in subsequent lines of therapy. It is likely that OS with D-Rd would be even longer in the current multiple myeloma treatment landscape with the use of novel therapies, such as bispecific antibodies and CAR-T therapy, in subsequent lines of therapy.

The observed OS benefit should be interpreted in the context of therapies administered following first-line treatment. The addition of daratumumab to proteasome inhibitors and immunomodulatory drugs, including and beyond lenalidomide, has been shown to significantly improve PFS, and, in some cases, OS, even in later lines of therapy [24–26]. In MAIA, not all patients in the Rd arm who received subsequent treatment received subsequent daratumumab. Nevertheless, given the high attrition rate—particularly among older patients ineligible for intensive therapy [27]—the OS benefit reported for the current study, together with the pronounced improvement in PFS observed previously [17] and potential reduction in disease-related morbidity, strongly supports the use of daratumumab in the frontline setting.

After the completion of this final analysis, no new safety concerns related to death were found for D-Rd. While the rates of adverse events as the primary cause of death were similar with D-Rd and Rd, the rates of death due to disease progression were lower with D-Rd versus Rd. A slightly higher rate of neoplasms as the primary cause of death was observed with D-Rd compared with Rd, which may be attributable to a longer duration of lenalidomide exposure in the D-Rd group compared with the Rd group; further investigation, potentially via a competing risks analysis that treats deaths due to other primary causes as competing events and takes confounding risk factors into account or an analysis of exposure-adjusted incidence rate of deaths due to neoplasms, is warranted. However, this was a long-term follow-up survival analysis, and adverse events were not collected per protocol.

The long-term benefits with D-Rd treatment discussed herein, at >7 years’ median follow-up of MAIA, were consistent with those observed at an earlier clinical cutoff. At a median follow-up of 64.5 months, PFS (HR, 0.55; 95% CI, 0.45–0.67; *P* < 0.0001) and OS benefits (HR, 0.66; 95% CI, 0.53–0.83; *P* = 0.0003) were observed with the addition of daratumumab to Rd compared with Rd alone. Median PFS was reached in the D-Rd group and was 61.9 months versus 34.4 months in the Rd group [17]. Moreover, the rate of sustained MRD negativity (10^–5^) lasting ≥18 months was higher with D-Rd versus Rd (16.8% vs 3.3%, respectively; *P* < 0.0001), further substantiating the observed OS and PFS benefit. Additionally, greater and more rapid improvements in quality-of-life outcomes, such as pain and global health status, were reported by patients in the D-Rd group versus the Rd group [28].

Cross-study comparisons should be considered with caution due to differences in study design and patient populations, but these results of the final analysis of MAIA complement and compare favorably to those of other long-term analyses of clinical studies in patients with NDMM. In the phase 3 ALCYONE study in transplant-ineligible patients with NDMM, at a median follow-up of 86.7 months, daratumumab combined with bortezomib, melphalan, and prednisone (D-VMP) prolonged OS versus bortezomib, melphalan, and prednisone (VMP) alone (median OS, 83.0 vs 53.6 months; HR, 0.65; 95% CI, 0.53–0.80; *P* < 0.0001). Notably, this HR is similar to that observed in the current study, with a median OS of 90.3 months with D-Rd versus 64.1 months with Rd (HR, 0.67; 95% CI, 0.55–0.82; *P* < 0.0001), despite the non–head-to-head nature of these comparisons. Furthermore, analysis of OS in prespecified subgroups demonstrated consistent superiority of D-VMP over VMP across subgroups. The OS HR point estimate favored D-VMP over VMP among both patients who achieved MRD negativity (HR, 0.60; 95% CI, 0.31–1.14) and those who did not achieve MRD negativity (HR, 0.77; 95% CI, 0.62–0.95) [29]. The phase 3 SWOG S0777 trial evaluated the efficacy and safety of bortezomib, lenalidomide, and dexamethasone (VRd) versus Rd alone in patients with NDMM without intent for immediate transplant. At a median follow-up of 84 months, median PFS was 41 months for VRd versus 29 months for Rd (stratified HR, 0.742; 96% Wald CI, 0.594–0.828; 1-sided stratified log-rank *P* = 0.003); median OS was not reached for VRd versus 69 months for Rd (stratified HR, 0.709; 96% Wald CI, 0.543–0.926; 2-sided stratified *P* = 0.0114) [30]. Analysis of OS based on age demonstrated therapeutic benefit of VRd over Rd both in patients aged <65 years and ≥65 years. It is important to note that median age was higher in MAIA than in SWOG S0777 (73 vs 63 years), more patients in MAIA were aged ≥65 years than in SWOG S0777 (99% vs 43%), and all patients in MAIA were ineligible for transplant while 32% of patients in SWOG S0777 were not intended for transplant [30, 31].

Recently, the phase 3 IMROZ trial evaluated the efficacy and safety of isatuximab combined with VRd (Isa-VRd) versus VRd alone in transplant-ineligible patients with NDMM. At a median follow-up of 59.7 months, the risk of disease progression or death was reduced with Isa-VRd versus VRd alone (HR, 0.60; 98.5% CI, 0.41–0.88; *P* < 0.001) [32]. Additionally, the phase 3 CEPHEUS trial investigated the efficacy and safety of daratumumab combined with VRd (D-VRd) versus VRd alone in transplant-ineligible or transplant-deferred patients with NDMM. At a median follow-up of 58.7 months, the risk of disease progression or death was reduced for D-VRd versus VRd alone (HR, 0.57; 95% CI, 0.41–0.79; *P* = 0.0005) [33]. The IMROZ and CEPHEUS trials both demonstrated OS HRs that trended in favor of the quadruplet therapies compared with VRd alone, with OS follow-up still ongoing for these studies [32, 33]. The MAIA study uniquely provides long-term follow-up data on OS, demonstrating a >2-year benefit in median OS with D-Rd versus Rd at the time of the final analysis. The totality of data from MAIA has established D-Rd as a standard of care for transplant-ineligible patients with NDMM, with consistent benefits compared with Rd observed across age, frailty, and risk status subgroups [13, 17, 23, 34]. While growing evidence also supports the efficacy benefits of quadruplet therapies for this patient population [32, 33], it is important to balance the potential for greater efficacy with quadruplet therapy against the improved tolerability observed with triplet D-Rd therapy. Choice of first-line therapy ultimately needs to be determined for each individual patient based on their treatment goals and ability to tolerate the addition of a proteasome inhibitor [33].

This study had some potential limitations. First, this study had an open-label design, which might have led to a bias for early patient withdrawals in the Rd group. Another potential limitation was that the prespecified subgroup analysis of OS included some subgroups with small sample sizes (eg, non-White patients, patients with impaired baseline hepatic function, and patients with high cytogenetic risk), and thus results in these subgroups should be interpreted with caution. According to the protocol amendment, updated data were not collected on efficacy endpoints beyond survival or on adverse events, and missing data are always a limitation for any clinical trial. Additionally, data collection was focused on subsequent antimyeloma therapy, PFS on the next line of therapy, second primary malignancies, and survival; safety data captured during this final analysis of MAIA were limited to death-related data only.

In summary, in this final follow-up of the MAIA study, at a median follow-up of approximately 7.5 years, median OS was finally reached in the D-Rd group and was 90.3 months (>7.5 years), setting a new benchmark for the population of transplant-ineligible patients with NDMM. D-Rd continued to demonstrate a clinical OS benefit versus Rd alone and prolonged median time to subsequent antimyeloma treatment. In addition, almost 30% of patients treated with Rd received daratumumab-based regimens as subsequent antimyeloma therapy, further emphasizing daratumumab as a standard of care for transplant-ineligible patients with multiple myeloma. With this long-term follow-up of MAIA, the OS benefit observed with the addition of daratumumab to the Rd standard-of-care regimen continues to support the frontline use of D-Rd to maximize survival in transplant-ineligible patients with NDMM.

## Supplementary information

Supplementary Appendix

## Data Availability

The data sharing policy of Johnson & Johnson is available at https://innovativemedicine.jnj.com/our-innovation/clinical-trials/transparency. As noted on this site, requests for access to the study data can be submitted through the Yale Open Data Access (YODA) Project site at http://yoda.yale.edu.
