# Supplementary material for: Daratumumab, lenalidomide, and dexamethasone in transplant-ineligible newly diagnosed multiple myeloma: MAIA final survival analysis
Source: Leukemia. 2026 Aug 24;40(11):2530–9. doi: 10.1038/s41375-026-03097-9 (PMC13713557; doi:10.1038/s41375-026-03097-9)
Supplement: Supplementary file 1 [file 41375_2026_3097_MOESM1_ESM.pdf]

Supplementary Appendix

Supplement to: Facon T, et al. Daratumumab, lenalidomide, and dexamethasone in transplant-ineligible newly diagnosed multiple myeloma: MAIA final survival analysis

Table of Contents

Additional methods ... 2

Supplementary Table 1. Ethics committees/institutional review boards in the MAIA study 3

Supplementary Table 2. Demographic and baseline disease characteristics in the ITT population ... 30

Supplementary Table 3. Summary of patients who did not receive subsequent antimyeloma therapy in the ITT population ... 32

Supplementary Figure 1. OS by MRD (10^{-5}) status and treatment group in the ITT population. ... 33

Supplementary Figure 2. OS by sustained MRD negativity (10^{-5}) status and treatment group in the ITT population. ... 34

Supplementary Figure 3. Best response to first subsequent antimyeloma therapy in the ITT population. ... 35

References ... 36

Additional methods

Definitions of efficacy endpoints
Progression-free survival (PFS) was defined as the duration from date of randomization to either progressive disease, according to the International Myeloma Working Group (IMWG response criteria [1,2], or death, whichever occurred first. Patients who started subsequent antimyeloma therapies for multiple myeloma without disease progression were censored at the last disease assessment before the start of subsequent therapies. Patients who withdrew consent from the study before disease progression were censored at the last disease assessment before withdrawal of consent. Patients who were lost to follow-up were censored at the last disease assessment before patients were lost to follow-up. Patients who had not progressed and were still alive at the cutoff date for analysis were censored at the last disease assessment. Patients without any post-baseline disease assessment were censored at randomization.

Complete response or better (≥CR) rate was defined as the proportion of patients with a response of CR or better (ie, CR or stringent CR [sCR]) based on the computerized algorithm, according to IMWG response criteria, during or after study treatment.

Very good partial response or better (≥VGPR) rate was defined as the proportion of patients with a response of VGPR or better (ie, VGPR, CR, or sCR) based on the computerized algorithm, according to IMWG response criteria, during or after study treatment.

Minimal residual disease (MRD)-negativity rate was defined as the proportion of patients who had negative MRD at any time point after the date of randomization and prior to subsequent antimyeloma therapy. MRD positive patients include patients for which all tested results were found to be MRD positive, or indeterminate, or unavailable (calibration failure or missing).

Overall response rate (ORR) was defined as the proportion of patients who achieved a partial response (PR) or better (ie, PR, VGPR, CR, or sCR) based on the computerized algorithm, according to IMWG response criteria, during or after study treatment.

Overall survival (OS) was defined as the date of randomization to the date of death due to any cause. Patients lost to follow-up were censored at the time of lost to follow-up. Patients who were still alive at the clinical cut-off date for the analysis were censored at the last known alive date. The date of last known alive date was determined by the maximum collection/assessment date from among selected data domains within the clinical database.

Time to subsequent antimyeloma treatment was defined as the time from randomization to the start of subsequent antimyeloma treatment. Death due to progressive disease without start of subsequent therapy was considered as an event. Patients who withdrew consent from the study, were lost to follow-up, or died due to causes other than disease progression were censored at the date of death or the last date known to be alive.

Supplementary Table 1. Ethics committees/institutional review boards in the MAIA study

Region	Name/Address	Site	Approval Date
AUSTRALIA	Hollywood Private Hospital Human Research Ethics Committee<br>101 Monash Avenue, Nedlands WA 6009, Australia	610801<br>101 Monash Avenue, 6009, NEDLANDS	Region: NA<br>Site, Central: 19-Feb-2016<br>Site, Local: 19-Feb-2016
AUSTRALIA	St Vincent's Hospital (Melbourne) Human Research Ethics Committee D<br>41 Victoria Parade, Fitzroy VIC 3065, Australia	610802<br>28 Woodville Road, South Australia, 5011, Woodville	Region: NA<br>Site, Central: 03-Jun-2015<br>Site, Local: 07-Dec-2015
AUSTRALIA	St Vincent's Hospital (Melbourne) Human Research Ethics Committee D<br>41 Victoria Parade, Fitzroy VIC 3065, Australia	610803<br>5 Arnold St, Victoria, 3128, Box Hill	Region: NA<br>Site, Central: 03-Jun-2015<br>Site, Local: 03-Jun-2015
AUSTRALIA	St Vincent's Hospital (Melbourne) Human Research Ethics Committee D<br>41 Victoria Parade, Fitzroy VIC 3065, Australia	610804<br>176 Furlong Road, Victoria, 3021, St Albans	Region: NA<br>Site, Central: 03-Jun-2015<br>Site, Local: 25-Aug-2015
AUSTRALIA	St Vincent's Hospital (Melbourne) Human Research Ethics Committee D<br>41 Victoria Parade, Fitzroy VIC 3065, Australia	610805<br>30 Gray Street, New South Wales, 2217, Kogarah	Region: NA<br>Site, Central: 03-Jun-2015<br>Site, Local: 08-Jul-2015
AUSTRALIA	St Vincent's Hospital (Melbourne) Human Research Ethics Committee D<br>41 Victoria Parade, Fitzroy VIC 3065, Australia	610806<br>199 Ipswich Road Level 3, R Block, Building 1, Queensland, 4102, Woolloongabba	Region: NA<br>Site, Central: 15-Jul-2015<br>Site, Local: 24-Nov-2015

AUSTRALIA	Bellberry Limited Human Research Ethics Committee<br>129 Glen Osmond Road, Eastwood SA 5063, Australia	610808<br>Tennyson Centre Level 1, 520 South Rd, South Australia, 5037, Kurralta Park	Region: NA<br>Site, Central: 20-Jul-2015<br>Site, Local: 20-Jul-2015
AUSTRALIA	St Vincent's Hospital (Melbourne) Human Research Ethics Committee D<br>41 Victoria Parade, Fitzroy VIC 3065, Australia	610809<br>41 Victoria Parade, 3065, Fitzroy	Region: NA<br>Site, Central: 03-Jun-2015<br>Site, Local: 14-Jul-2015
AUSTRALIA	St Vincent's Hospital (Melbourne) Human Research Ethics Committee D<br>41 Victoria Parade, Fitzroy VIC 3065, Australia	610810<br>Corner of Edith & Platt Streets, New South Wales, 2298, Waratah	Region: NA<br>Site, Central: 03-Jun-2015<br>Site, Local: 24-Jul-2015
AUSTRIA	Ethikkommission der Stadt Wien TownTown, Thomas-Klestil-Platz 8, A-1030 Wien AUSTRIA	430801<br>Heinrich-Collin-Straße 30, Wien, 1140, Wien	Region: 26-May-2015<br>Site, Central: 26-May-2015<br>Site, Local: 26-May-2015
AUSTRIA	Ethikkommission für das Bundesland Salzburg Pfeifergasse 7; A-5020 Salzburg AUSTRIA	430802<br>Landeskrankenhaus Salzburg Universitaetsklinik fuer Innere Medizin III, Salzburg, 5020, Salzburg	Region: 26-May-2015<br>Site, Central: 26-May-2015<br>Site, Local: 26-May-2015
AUSTRIA	Ethikkommission der Medizinischen Universität Innsbruck Anichstraße 35; A-6020 Innsbruck AUSTRIA	430803<br>Anichstraße 35, 6020, Innsbruck	Region: 26-May-2015<br>Site, Central: 26-May-2015<br>Site, Local: 26-May-2015
AUSTRIA	Ethikkommission der Medizinischen Universität Wien Borschkegasse 8b/E06 A-1090 Wien AUSTRIA	430805<br>Wahringer Gurtel 18-20<br>1090 Wein, Vienna	

BELGIUM	Comité d'Ethique Hospitalo-Facultaire<br>CHU Liège<br>Domaine Universitaire du Sart Tilman - B35<br>4000 Liège	320802<br>Domaine du Sart-Tilman B35<br>4000 Liège	Region: 08-Apr-2016<br>Site, Central: 08-Apr-2016<br>Site, Local: 08-Apr-2016
BELGIUM	Commissie Medische Ethiek van Universitaire Ziekenhuizen K.U. Leuven<br>Campus Gasthuisberg E330, Herestraat 49 - 3000 Leuven - Belgium	320803<br>Herestraat 49,<br>Vlaams Brabant,<br>3000, Leuven	Region: 08-Apr-2016<br>Site, Central: 08-Apr-2016<br>Site, Local: 08-Apr-2016
BELGIUM	Comité d'éthique des centres hospitalier Jolimont-Lobbes et Nivelles- l'ubize<br>Hôpital de Jolimont<br>Rue Ferrrer 158<br>7100 Haine Saint Paul<br>Belgium	320805<br>Rue Ferrer, 159,<br>Hainaut, 7100,<br>Haine-Saint-Paul	Region: 08-Apr-2016<br>Site, Central: 08-Apr-2016<br>Site, Local: 08-Apr-2016
CANADA	Comite d'ethique de la recherche du CHUM Tour Viger, Pavillon R, 900 rue St-Denis, 3e etage	110801<br>1560 Sherbrooke East<br>Pav. L.C. Simard,<br>H2L4M1, Montreal	Region: NA<br>Site, Central: NA<br>Site, Local: 27-Oct-2015
CANADA	Health Research Ethics Board of Alberta-Cancer Committee (HREBA-CC) 1500, 10104 - 103 Avenue NW<br>Edmonton, Alberta, T5J 4A7, CANADA	110802<br>11560 University<br>Avenue NW,<br>Edmonton	Region: NA<br>Site, Central: NA<br>Site, Local: 27-Aug-2015
CANADA	Health Research Ethics Board of Alberta-Cancer Committee (HREBA-CC) 1500, 10104 - 103 Avenue NW<br>Edmonton, Alberta, T5J 4A7, CANADA	110803<br>1403 -29th St NW,<br>Alberta, T2N 2T9,<br>Calgary	Region: NA<br>Site, Central: NA<br>Site, Local: 13-Nov-2015
CANADA	Comite d'ethique de la recherche du CHUM Tour Viger, Pavillon R, 900 rue St-Denis, 3e etage	110804<br>1401, 18e Rue,<br>bureau N-1760, G1J<br>1Z4, Quebec, PQ	Region: NA<br>Site, Central: NA<br>Site, Local: 27-Nov-2015

CANADA	University Health Network Research, Ethics Board<br>700 University Avenue 10Th Floor South, Room 1056<br>Toronto Ontario M5G 1Z5	110805<br>610 University Avenue<br>Toronto-Ontario<br>M5G 2M9	Region: NA<br>Site, Central: NA<br>Site, Local:
CANADA	Comite d'ethique de la recherche du CHUM Tour Viger, Pavillon R, 900 rue St-Denis, 3e etage	110807<br>1001 Decarie Blvd. room: D02.6720, Quebec, H4A-3J1, Montreal	Region: NA<br>Site, Central: NA<br>Site, Local: 14-Jan-2016
CANADA	Comite d'ethique de la recherche du CHUM Tour Viger, Pavillon R, 900 rue St-Denis, 3e etage	110808<br>3120, Boulevard Tachereau, Quebec, J4V 2H1, Greenfield Park	Region: NA<br>Site, Central: NA<br>Site, Local: 23-Dec-2015
CANADA	Nova Scotia Health Authority Research Ethics Board<br>Centre for Clinical Research<br>5790 University Avenue - Room 118<br>Halifax, N.S.<br>Canada B3H 1V7	110809<br>1278 South Park Street, Nova Scotia, B3H 2Y9, Halifax	Region: NA / Site, Central: NA / Site, Local: 03-Mar-2016
DENMARK	The Regional Scientific Ethical Committees for Southern Denmark<br>Regionshuset, Damhaven 12, 7100 Vejle, Denmark	450801<br>Beriderbakken 4<br>Medicinsk Afdeling, A140 Hæmatologisk sengeafsnit, DK-7100, Vejle	Region: 30-Jun-2015<br>Site, Central: 30-Jun-2015<br>Site, Local: N/A
DENMARK	The Regional Scientific Ethical Committees for Southern Denmark<br>Regionshuset, Damhaven 12, 7100 Vejle, Denmark	450802<br>Kløvervænget 10, 12th Floor, South Denmark, 5000, Odense	Region: 30-Jun-2015<br>Site, Central: 30-Jun-2015<br>Site, Local: N/A

DENMARK	The Regional Scientific Ethical Committees for Southern Denmark<br>Regionshuset, Damhaven 12, 7100 Vejle, Denmark	450803<br>Palle Juul-Jensens Boulevard 99<br>Indgang C, plan 1,<br>Krydspunkt C116,<br>Central Jutland, 8200, Aarhus N	Region: 30-Jun-2015<br>Site, Central: 30-Jun-2015<br>Site, Local: N/A
FRANCE	CHRU LILLE C.P.P. Nord-Ouest IV - CHRU LILLE<br>Bâtiment ex-USN B<br>6 rue du Professeur Laguesse<br>59037 Lille Cedex France	330801<br>Avenue de la Cote de Nacre 14000 Caen	Region: 07-Jul-2015<br>Site, Central: 07-Jul-2015<br>Site, Local: NA
FRANCE	CHRU LILLE C.P.P. Nord-Ouest IV - CHRU LILLE<br>Bâtiment ex-USN B<br>6 rue du Professeur Laguesse<br>59037 Lille Cedex France	330802<br>165 chemin du grand Revoyet 165 chemin du Grand Revoyet Hématologie, 69495, Pierre-Benite	Region: 07-Jul-2015<br>Site, Central: 07-Jul-2015<br>Site, Local: NA
FRANCE	CHRU LILLE C.P.P. Nord-Ouest IV - CHRU LILLE<br>Bâtiment ex-USN B<br>6 rue du Professeur Laguesse<br>59037 Lille Cedex France	330803<br>Rue Michel Polonowski, 59037, LILLE Cedex	Region: 07-Jul-2015<br>Site, Central: 07-Jul-2015<br>Site, Local: NA
FRANCE	CHRU LILLE C.P.P. Nord-Ouest IV - CHRU LILLE<br>Bâtiment ex-USN B<br>6 rue du Professeur Laguesse<br>59037 Lille Cedex France	330804<br>13 Avenue de L'interne J. Loeb, Pyrénées-Atlantiques, 64100, Bayonne	Region: 07-Jul-2015<br>Site, Central: 07-Jul-2015<br>Site, Local: NA
FRANCE	CHRU LILLE C.P.P. Nord-Ouest IV - CHRU LILLE<br>Bâtiment ex-USN B<br>6 rue du Professeur Laguesse<br>59037 Lille Cedex France	330805<br>2, Boulevard Tonnellé, Centre, 37044, Tours	Region: 07-Jul-2015<br>Site, Central: 07-Jul-2015<br>Site, Local: NA

FRANCE	CHRU LILLE C.P.P. Nord-Ouest IV - CHRU LILLE<br>Bâtiment ex-USN B<br>6 rue du Professeur Laguesse<br>59037 Lille Cedex France	330806<br>10 Rue Marcel Proust, Côtes-d'Armor, 22027, Saint Brieuc	Region: 07-Jul-2015<br>Site, Central: 07-Jul-2015<br>Site, Local: NA
FRANCE	CHRU LILLE C.P.P. Nord-Ouest IV - CHRU LILLE<br>Bâtiment ex-USN B<br>6 rue du Professeur Laguesse<br>59037 Lille Cedex France	330807<br>80, avenue Augustin FLICHE, 34295, Montpellier Cedex 5	Region: 07-Jul-2015<br>Site, Central: 07-Jul-2015<br>Site, Local: NA
FRANCE	CHRU LILLE C.P.P. Nord-Ouest IV - CHRU LILLE<br>Bâtiment ex-USN B<br>6 rue du Professeur Laguesse<br>59037 Lille Cedex France	330808<br>Boulevard de Belfort BP 387, Nord, 59020, LILLE CEDEX	Region: 07-Jul-2015<br>Site, Central: 24-Sep-2015<br>Site, Local: NA
FRANCE	CHRU LILLE C.P.P. Nord-Ouest IV - CHRU LILLE<br>Bâtiment ex-USN B<br>6 rue du Professeur Laguesse<br>59037 Lille Cedex France	330809<br>51 av. du Maréchal de lattre de Tassigny Unité Hémopathies Lymphoïdes, Île-de-France, 94010, Créteil	Region: 07-Jul-2015<br>Site, Central: 24-Sep-2015<br>Site, Local: NA
FRANCE	CHRU LILLE C.P.P. Nord-Ouest IV - CHRU LILLE<br>Bâtiment ex-USN B<br>6 rue du Professeur Laguesse<br>59037 Lille Cedex France	330810<br>2, Avenue Martin Luther King, Haute-Vienne, 87042, Limoges	Region: 07-Jul-2015<br>Site, Central: 07-Jul-2015<br>Site, Local: NA
FRANCE	CHRU LILLE C.P.P. Nord-Ouest IV - CHRU LILLE<br>Bâtiment ex-USN B<br>6 rue du Professeur Laguesse<br>59037 Lille Cedex France	330811<br>80 Avenue Georges Pompidou - CS 61205<br>24000 PERIGUEUX	Region: 07-Jul-2015<br>Site, Central: 07-Jul-2015<br>Site, Local: NA

FRANCE	CHRU LILLE C.P.P. Nord-Ouest IV -<br>CHRU LILLE<br>Bâtiment ex-USN B<br>6 rue du Professeur Laguesse<br>59037 Lille Cedex France	330812<br>Centre François Magendie Avenue de Magellan, Aquitaine, 33608, Pessac	Region: 07-Jul-2015<br>Site, Central: 07-Jul-2015<br>Site, Local: NA
FRANCE	CHRU LILLE C.P.P. Nord-Ouest IV -<br>CHRU LILLE<br>Bâtiment ex-USN B<br>6 rue du Professeur Laguesse<br>59037 Lille Cedex France	330813<br>Boulevard Stephane Moreau, Vendée, 85925, La Roche Sur Yon	Region: 07-Jul-2015<br>Site, Central: 24-Sep-2015<br>Site, Local: NA
FRANCE	CHRU LILLE C.P.P. Nord-Ouest IV -<br>CHRU LILLE<br>Bâtiment ex-USN B<br>6 rue du Professeur Laguesse<br>59037 Lille Cedex France	330814<br>Place Victor Pauchet, Somme, 80054, Amiens	Region: 07-Jul-2015<br>Site, Central: 07-Jul-2015<br>Site, Local: NA
FRANCE	CHRU LILLE C.P.P. Nord-Ouest IV -<br>CHRU LILLE<br>Bâtiment ex-USN B<br>6 rue du Professeur Laguesse<br>59037 Lille Cedex France	330815<br>Place Alexis Ricordeau Merge with Master, Loire-Atlantique, 44000, Nantes	Region: 07-Jul-2015<br>Site, Central: 24-Sep-2015<br>Site, Local: NA
FRANCE	CHRU LILLE C.P.P. Nord-Ouest IV -<br>CHRU LILLE<br>Bâtiment ex-USN B<br>6 rue du Professeur Laguesse<br>59037 Lille Cedex France	330816<br>20 Bd Guillauot, BP 70555, 56017, Vannes	Region: 07-Jul-2015<br>Site, Central: 24-Sep-2015<br>Site, Local: NA
FRANCE	CHRU LILLE C.P.P. Nord-Ouest IV -<br>CHRU LILLE<br>Bâtiment ex-USN B<br>6 rue du Professeur Laguesse<br>59037 Lille Cedex France	330817<br>177 Rue Versailles, 78157, Le Chesnay Cedex	Region: 07-Jul-2015<br>Site, Central: 24-Sep-2015<br>Site, Local: NA

FRANCE	CHRU LILLE C.P.P. Nord-Ouest IV - CHRU LILLE<br>Bâtiment ex-USN B<br>6 rue du Professeur Laguesse<br>59037 Lille Cedex France	330818<br>184 rue du Faubourg Saint Antoine, 75571, Paris cedex 12	Region: 07-Jul-2015<br>Site, Central: 07-Jul-2015<br>Site, Local: NA
FRANCE	CHRU LILLE C.P.P. Nord-Ouest IV - CHRU LILLE<br>Bâtiment ex-USN B<br>6 rue du Professeur Laguesse<br>59037 Lille Cedex France	330819<br>29 Avenue Pierre Mendes, Seine-Maritime, 76290, Montvilliers	Region: 07-Jul-2015<br>Site, Central: 24-Sep-2015<br>Site, Local: NA
FRANCE	CHRU LILLE C.P.P. Nord-Ouest IV - CHRU LILLE<br>Bâtiment ex-USN B<br>6 rue du Professeur Laguesse<br>59037 Lille Cedex France	330820<br>108 avenue Albert Raimond, 42270, Saint Priest en Jarez	Region: 07-Jul-2015<br>Site, Central: 24-Sep-2015<br>Site, Local: NA
FRANCE	CHRU LILLE C.P.P. Nord-Ouest IV - CHRU LILLE<br>Bâtiment ex-USN B<br>6 rue du Professeur Laguesse<br>59037 Lille Cedex France	330821<br>20 Avenue Du Dr Rene Laennec Merge with Master, Haut-Rhin, 68070, Mulhouse	Region: 07-Jul-2015<br>Site, Central: 24-Sep-2015<br>Site, Local: NA
FRANCE	CHRU LILLE C.P.P. Nord-Ouest IV - CHRU LILLE<br>Bâtiment ex-USN B<br>6 rue du Professeur Laguesse<br>59037 Lille Cedex France	330822<br>Avenue du Général Koenig cedex, Marne, 51092, Reims	Region: 07-Jul-2015<br>Site, Central: 24-Sep-2015<br>Site, Local: NA
FRANCE	CHRU LILLE C.P.P. Nord-Ouest IV - CHRU LILLE<br>Bâtiment ex-USN B<br>6 rue du Professeur Laguesse<br>59037 Lille Cedex France	330823<br>47-82 route de l'hôpital, Île-de-France, 75013, Paris	Region: 07-Jul-2015<br>Site, Central: 24-Sep-2015<br>Site, Local: NA

FRANCE	CHRU LILLE C.P.P. Nord-Ouest IV -<br>CHRU LILLE<br>Bâtiment ex-USN B<br>6 rue du Professeur Laguesse<br>59037 Lille Cedex France	330824<br>Rue d'Amiens Merge with Master - CS 11516 Haute-Normandie, 76038, Rouen	Region: 07-Jul-2015<br>Site, Central: 24-Sep-2015<br>Site, Local: NA
FRANCE	CHRU LILLE C.P.P. Nord-Ouest IV -<br>CHRU LILLE<br>Bâtiment ex-USN B<br>6 rue du Professeur Laguesse<br>59037 Lille Cedex France	330825<br>130 Avenue Louis Herbeaux Bp 6 367 BP 6-367, Nord, 59385, Dunkerque	Region: 07-Jul-2015<br>Site, Central: 07-Jul-2015<br>Site, Local: NA
FRANCE	CHRU LILLE C.P.P. Nord-Ouest IV -<br>CHRU LILLE<br>Bâtiment ex-USN B<br>6 rue du Professeur Laguesse<br>59037 Lille Cedex France	330826<br>1 Avenue De Molière, 67098, Strasbourg Cedex	Region: 07-Jul-2015<br>Site, Central: 24-Sep-2015<br>Site, Local: NA
FRANCE	CHRU LILLE C.P.P. Nord-Ouest IV -<br>CHRU LILLE<br>Bâtiment ex-USN B<br>6 rue du Professeur Laguesse<br>59037 Lille Cedex France	330827<br>hematologie CS 10217, Isère, 38043, grenoble cedex 9	Region: 07-Jul-2015<br>Site, Central: 07-Jul-2015<br>Site, Local: NA
FRANCE	CHRU LILLE C.P.P. Nord-Ouest IV -<br>CHRU LILLE<br>Bâtiment ex-USN B<br>6 rue du Professeur Laguesse<br>59037 Lille Cedex France	330828<br>1 avenue Irène Joliot-Curie, 31059, Toulouse Cedex 9	Region: 07-Jul-2015<br>Site, Central: 07-Jul-2015<br>Site, Local: NA
FRANCE	CHRU LILLE C.P.P. Nord-Ouest IV -<br>CHRU LILLE<br>Bâtiment ex-USN B<br>6 rue du Professeur Laguesse<br>59037 Lille Cedex France	330829<br>20 Av Du Languedoc Bp 49954 BP 49954 Languedoc-Roussillon, 66046, Perpignan	Region: 07-Jul-2015<br>Site, Central: 24-Sep-2015<br>Site, Local: NA

FRANCE	CHRU LILLE C.P.P. Nord-Ouest IV - CHRU LILLE<br>Bâtiment ex-USN B<br>6 rue du Professeur Laguesse<br>59037 Lille Cedex France	330830<br>149 rue de Sèvres Merge with Master - Île-de-France, 75015, Paris	Region: 07-Jul-2015<br>Site, Central: 24-Sep-2015<br>Site, Local: NA
FRANCE	CHRU LILLE C.P.P. Nord-Ouest IV - CHRU LILLE<br>Bâtiment ex-USN B<br>6 rue du Professeur Laguesse<br>59037 Lille Cedex France	330831<br>18 Rue Victor Hugo, 72000, Le Mans	Region: 07-Jul-2015<br>Site, Central: 07-Jul-2015<br>Site, Local: NA
FRANCE	CHRU LILLE C.P.P. Nord-Ouest IV - CHRU LILLE<br>Bâtiment ex-USN B<br>6 rue du Professeur Laguesse<br>59037 Lille Cedex France	330832<br>CHRU Hôpital Sud, 35203, RENNES	Region: 07-Jul-2015<br>Site, Central: 07-Jul-2015<br>Site, Local: NA
FRANCE	CHRU LILLE C.P.P. Nord-Ouest IV - CHRU LILLE<br>Bâtiment ex-USN B<br>6 rue du Professeur Laguesse<br>59037 Lille Cedex France	330833<br>27 Rue Du Faubourg Saint-Jacques, Paris, 75014, Paris	Region: 07-Jul-2015<br>Site, Central: 30-Sep-2015<br>Site, Local: NA
FRANCE	CHRU LILLE C.P.P. Nord-Ouest IV - CHRU LILLE<br>Bâtiment ex-USN B<br>6 rue du Professeur Laguesse<br>59037 Lille Cedex France	330834<br>1 Place Lucie et Raymond Aubrac, 63000, Clermont Ferrand	Region: 07-Jul-2015<br>Site, Central: 24-Sep-2015<br>Site, Local: NA
FRANCE	CHRU LILLE C.P.P. Nord-Ouest IV - CHRU LILLE<br>Bâtiment ex-USN B<br>6 rue du Professeur Laguesse<br>59037 Lille Cedex France	330835<br>4 Rue Larrey, Loire-Atlantique, 44093, Angers	Region: 07-Jul-2015<br>Site, Central: 24-Sep-2015<br>Site, Local: NA
FRANCE	CHRU LILLE C.P.P. Nord-Ouest IV - CHRU LILLE<br>Bâtiment ex-USN B	330836<br>1 avenue de l'hôpital, 74370, Metz Tessy	Region: 07-Jul-2015<br>Site, Central: 24-Sep-2015<br>Site, Local: NA

	6 rue du Professeur Laguesse<br>59037 Lille Cedex France		
FRANCE	CHRU LILLE C.P.P. Nord-Ouest IV -<br>CHRU LILLE<br>Bâtiment ex-USN B<br>6 rue du Professeur Laguesse<br>59037 Lille Cedex France	330837<br>6 Avenue De L'Ile De France Bp 79<br>Val-d'Oise, 95303, Pontoise	Region: 07-Jul-2015<br>Site, Central: 24-Sep-2015<br>Site, Local: NA
FRANCE	CHRU LILLE C.P.P. Nord-Ouest IV -<br>CHRU LILLE<br>Bâtiment ex-USN B<br>6 rue du Professeur Laguesse<br>59037 Lille Cedex France	330838<br>Rue du Morvan, 54500, Vandoeuvre les Nancy	Region: 07-Jul-2015<br>Site, Central: 24-Sep-2015<br>Site, Local: NA
FRANCE	CHRU LILLE C.P.P. Nord-Ouest IV -<br>CHRU LILLE<br>Bâtiment ex-USN B<br>6 rue du Professeur Laguesse<br>59037 Lille Cedex France	330839<br>151 route Saint Antoine de Ginestiere, Alpes-Maritimes, 6202, Nice	Region: 07-Jul-2015<br>Site, Central: 24-Sep-2015<br>Site, Local: NA
FRANCE	CHRU LILLE C.P.P. Nord-Ouest IV -<br>CHRU LILLE<br>Bâtiment ex-USN B<br>6 rue du Professeur Laguesse<br>59037 Lille Cedex France	330840<br>1, Avenue Claude Vellefaux, Île-de-France, 75010, PARIS	Region: 07-Jul-2015<br>Site, Central: 24-Sep-2015<br>Site, Local: NA
FRANCE	CHRU LILLE C.P.P. Nord-Ouest IV -<br>CHRU LILLE<br>Bâtiment ex-USN B<br>6 rue du Professeur Laguesse<br>59037 Lille Cedex France	330841<br>232, boulevard de Sainte Marguerite, 3273, Marseille	Region: 07-Jul-2015<br>Site, Central: 06-May-2016<br>Site, Local: NA
FRANCE	CHRU LILLE C.P.P. Nord-Ouest IV -<br>CHRU LILLE<br>Bâtiment ex-USN B	330842<br>1 rue de la Marne<br>SAINT-MALO Cedex	

	6 rue du Professeur Laguesse<br>59037 Lille Cedex France		
FRANCE	CHRU LILLE C.P.P. Nord-Ouest IV -<br>CHRU LILLE<br>Bâtiment ex-USN B<br>6 rue du Professeur Laguesse<br>59037 Lille Cedex France	330844<br>1 Avenue Michel De L Hospital, 02321, Saint Quentin Cedex	Region: 07-Jul-2015<br>Site, Central: 06-May-2016<br>Site, Local: NA
FRANCE	CHRU LILLE C.P.P. Nord-Ouest IV -<br>CHRU LILLE<br>Bâtiment ex-USN B<br>6 rue du Professeur Laguesse<br>59037 Lille Cedex France	330845<br>2 rue de la Miletrie BP 577, 86021, Poitiers	Region: 07-Jul-2015<br>Site, Central: 16-Sep-2016<br>Site, Local: NA
FRANCE	CHRU LILLE C.P.P. Nord-Ouest IV -<br>CHRU LILLE<br>Bâtiment ex-USN B<br>6 rue du Professeur Laguesse<br>59037 Lille Cedex France	330847<br>2, boulevard Maréchal de Lattre de Tassigny, 21 034, Dijon	Region: 07-Jul-2015<br>Site, Central: 16-Sep-2016<br>Site, Local: NA
GERMANY	Technische Universität Dresden<br>Ethikkommission an der TU Dresden, Fetscherstraße 74, 01307 Dresden GERMANY	490801<br>Fetscherstr. 74, 01307, Dresden	Region: 31-Aug-2015<br>Site, Central: 31-Aug-2015<br>Site, Local: 31-Aug-2015
GERMANY	Ethikkommission der Landesärztekammer Rheinland-Pfalz Deutschhausplatz 3, 55116 Mainz GERMANY	490802<br>Langenbeckstraße 1, 55131, Mainz	Region: 31-Aug-2015<br>Site, Central: 31-Aug-2015<br>Site, Local: 31-Aug-2015
GERMANY	Ethikkommission der Landesärztekammer Rheinland-Pfalz Deutschhausplatz 3, 55116 Mainz GERMANY	490803<br>Neversstr. 5, Rheinland-Pfalz, 56068, Koblenz	Region: 31-Aug-2015<br>Site, Central: 31-Aug-2015<br>Site, Local: 31-Aug-2015
GERMANY	Ethikkommission der Medizinischen Fakultät Heidelberg, Alte Glockengießerei 11/1, 69115 Heidelberg GERMANY	490804<br>Im Neuenheimer Feld 410	Region: 31-Aug-2015<br>Site, Central: 31-Aug-2015<br>Site, Local: 31-Aug-2015

		Baden-Württemberg, 69120, Heidelberg	
GERMANY	Ethikkommission der Medizinischen Fakultät der Eberhard-Karls-Universität Tübingen Gartenstraße 47, 72074 Tübingen GERMANY	490805 Otfried-Müller-Str.10 Medizinische Klinik, Abteilung II - Haematologie, Onkologie, Klinische Immunologie , Baden-Württemberg, 72076, Tübingen	Region: 31-Aug-2015 Site, Central: 31-Aug-2015 Site, Local: 31-Aug-2015
GERMANY	Ethikkommission der Medizinischen Fakultät der Universität Duisburg-Essen Universitätsklinikum Essen, Robert-Koch-Str. 9-11; 45147 Essen GERMANY	490806 Hufelandstr. 55, Nordrhein-Westfalen, 45147, Essen	Region: 31-Aug-2015 Site, Central: 31-Aug-2015 Site, Local: 31-Aug-2015
GERMANY	Ethikkommission der Ärztekammer Nordrhein Tersteegenstraße 9, 40474 Düsseldorf GERMANY	490808 Pattbergstraße 1-3, Nordrhein-Westfalen, 45239, Koblenz	Region: 31-Aug-2015 Site, Central: 31-Aug-2015 Site, Local: 31-Aug-2015
GERMANY	Ethikkommission bei der Landesärztekammer Baden-Württemberg Liebknechtstraße 33 70565 Stuttgart GERMANY	490811 Auerbachstraße 110, Baden-Württemberg, 70376, Stuttgart	Region: 31-Aug-2015 Site, Central: 31-Aug-2015 Site, Local: 31-Aug-2015
GERMANY	Ethikkommission der Landesärztekammer Thüringen Im Semmicht 33, 07751 Jena GERMANY	490813 Robert Koch Allee 9 Klinik für Onkologie, Thüringen, 99437, Bad Berka	Region: 31-Aug-2015 Site, Central: 31-Aug-2015 Site, Local: 31-Aug-2015
GERMANY	Ethikkommission an der Medizinischen Fakultät der CAU Kiel, Haus U 27, Schwanenweg 20, 24105 Kiel GERMANY	490816 Schittenhelmstraße 12, 24105, Kiel	Region: 31-Aug-2015 Site, Central: 31-Aug-2015 Site, Local: 31-Aug-2015

GERMANY	Ethikkommission bei der Landesärztekammer Baden-Württemberg Liebknechtstraße 33<br>70565 Stuttgart GERMANY	490817<br>Q 5, 14 - 22 (7.OG), 68161, Mannheim	Region: 31-Aug-2015<br>Site, Central: 31-Aug-2015<br>Site, Local: 31-Aug-2015
GERMANY	Ethikkommission bei der Landesärztekammer Baden-Württemberg Liebknechtstraße 33<br>70565 Stuttgart GERMANY	490818<br>Klinikstr. 11, Baden-Württemberg, 78052, Villingen-Schwenningen	Region: 31-Aug-2015<br>Site, Central: 31-Aug-2015<br>Site, Local: 31-Aug-2015
GERMANY	Ethikkommission zur Beurteilung medizinischer Forschung am Menschen der Ärztekammer Niedersachsen<br>Berliner Allee 20;<br>30175 Hannover GERMANY	490821<br>Celler Strasse 38, Niedersachsen, 38114, Braunschweig	Region: 31-Aug-2015<br>Site, Central: 31-Aug-2015<br>Site, Local: 31-Aug-2015
GERMANY	Ethikkommission an der Medizinischen Fakultät der Universität Rostock St.-Georg-Str. 108, 18055 Rostock GERMANY	490822<br>Ernst-Heydemann-Str. 6<br>18057 Rostock	Region: 31-Aug-2015<br>Site, Central: 31-Aug-2015<br>Site, Local: 31-Aug-2015
IRELAND	Beaumont Hospital Ethics (Medical Research) Committee Beaumont Hospital, Dublin 9	353801<br>Newcastle Road, Galway	Region: 24-Apr-2015<br>Site, Central: NA<br>Site, Local: 12-Aug-2015
IRELAND	Beaumont Hospital Ethics (Medical Research) Committee Beaumont Hospital, Dublin 9	353802<br>Eccles Street 7, Dublin	Region: 24-Apr-2015<br>Site, Central: NA<br>Site, Local: 04-Feb-2016
ISRAEL	Helsinki Committee of Galilee Medical Center Meona Rd.,P.O. Box 21 Nahariya, 2210001, Israel,	972803<br>Meona Rd, HaZafon, 22100, Nahariya	Region: NA<br>Site, Central: NA<br>Site, Local: 27-Sep-2015
ISRAEL	Helsinki Committee (IRB) of Hadassah Medical Organization Kiryat Hadassah P.O. Box 12000, Jerusalem, 9112001, Israel	972804<br>Kiryat Hadassah, HaMerkaz, 91120, Jerusalem	Region: NA<br>Site, Central: NA<br>Site, Local: 19-Feb-2016

ISRAEL	Helsinki Committee of Rabin Medical Center, Beilinson Hospital 39 Jabotinski St., Petah Tikva, 4941492, Israel	972805<br>39 Jabotinski St., 49100, Petah Tikva	Region: NA<br>Site, Central: NA<br>Site, Local: 02-Sep-2015
ISRAEL	Helsinki Committee of Carmel Medical Center 7 Michal St., Haifa, 3436212, Israel	972807<br>Michal 7, 3436212, Haifa	Region: NA<br>Site, Central: NA<br>Site, Local: 24-Jul-2016
ITALY	Struttura per Comitato Etico Regione Toscana - Area Vasta Nord Ovest<br>Azienda Ospedaliero-Universitaria Pisana<br>Stabilimento di Santa Chiara<br>Via Roma, 67<br>56126 Pisa	390802<br>Viale G.B. Morgagni, 85, 50139, Firenze	Region: 27-Oct-2015<br>Site, Central: NA<br>Site, Local: 29Aug2016
ITALY	Struttura per Comitato Etico Regione Toscana - Area Vasta Nord Ovest<br>Azienda Ospedaliero-Universitaria Pisana<br>Stabilimento di Santa Chiara<br>Via Roma, 67<br>56126 Pisa	390804<br>Via Roma, 67, 56126, Pisa	Region: 27-Oct-2015<br>Site, Central: 27-Oct-2015<br>Site, Local: 27-Oct-2015
ITALY	Struttura per Comitato Etico Regione Toscana - Area Vasta Nord Ovest<br>Azienda Ospedaliero-Universitaria Pisana<br>Stabilimento di Santa Chiara<br>Via Roma, 67<br>56126 Pisa	390807<br>Via Gramsci 14, Parma, 43126, Parma	Region: 27-Oct-2015<br>Site, Central: NA<br>Site, Local: 28-Jul 2016
ITALY	Struttura per Comitato Etico Regione Toscana - Area Vasta Nord Ovest<br>Azienda Ospedaliero-Universitaria Pisana<br>Stabilimento di Santa Chiara<br>Via Roma, 67<br>56126 Pisa	390808<br>P.le dell'Umanesimo 10, 00144, Roma	Region: 27-Oct-2015<br>Site, Central: NA<br>Site, Local: 8Apr 2016
NETHERLANDS	METC Brabant Gebouw Hasseltveste, Dr. Deelenlaan 9, Tilburg, Noord-Brabant, 5042 AD, NETHERLANDS	310803<br>Daniel den Hoed Kliniek Groene	Region: 12-May-2015<br>Site, Central: 12-May-2015<br>Site, Local: 26-Aug-2015

		Hilledijk 301, Zuid-Holland, 3075 EA, Rotterdam	
NETHERLANDS	METC Brabant Gebouw Hasseltveste, Dr. Deelenlaan 9, Tilburg, Noord-Brabant, 5042 AD, NETHERLANDS	310804<br>Spaarnepoort 1, Noord-Holland, 2134 TM, Hoofddorp	Region: 12-May-2015<br>Site, Central: 12-May-2015<br>Site, Local: 02-Jul-2015
SWEDEN	Regionala Etikprövningsnämnden i Stockholm Tomtebodavägen 18A, Plan 3, 17165 Solna, Sweden, Christina Löfgren	460801<br>Sjukhusvägen 10, 97180, Luleå	Region: 11-Mar-2015<br>Site, Central: 11-Mar-2015<br>Site, Local: NA
SWEDEN	Regionala Etikprövningsnämnden i Stockholm Tomtebodavägen 18A, Plan 3, 17165 Solna, Sweden, Christina Löfgren	460802<br>Falu lasarett, 791 82, Falun	Region: 11-Mar-2015<br>Site, Central: 11-Mar-2015<br>Site, Local: NA
SWEDEN	Regionala Etikprövningsnämnden i Stockholm Tomtebodavägen 18A, Plan 3, 17165 Solna, Sweden, Christina Löfgren	460803<br>Skåne Universitetssjukhus Getingevägen 4, SE-221 85, Lund	Region: 11-Mar-2015<br>Site, Central: 11-Mar-2015<br>Site, Local: NA
SWEDEN	Regionala Etikprövningsnämnden i Stockholm Tomtebodavägen 18A, Plan 3, 17165 Solna, Sweden, Christina Löfgren	460804<br>Karolinska M54, 141 86, Stockholm	Region: 11-Mar-2015<br>Site, Central: 11-Mar-2015<br>Site, Local: NA
SWEDEN	Regionala Etikprövningsnämnden i Stockholm Tomtebodavägen 18A, Plan 3, 17165 Solna, Sweden, Christina Löfgren	460807<br>Bruna stråket 5, 5th floor, 41345, Göteborg	Region: 11-Mar-2015<br>Site, Central: 11-Mar-2015<br>Site, Local: NA
SWEDEN	Regionala Etikprövningsnämnden i Stockholm Tomtebodavägen 18A, Plan 3, 17165 Solna, Sweden, Christina Löfgren	460809<br>Hallands sjukhus, 301 85, Halmstad	Region: 11-Mar-2015<br>Site, Central: 05-May-2015<br>Site, Local: NA
UNITED KINGDOM	South West – Central Bristol South West REC Centre, Level 3, Block B, Whitefriars, Lewins Mead Bristol UNITED KINGDOM	440801<br>Oxford Road, M13 9WL, Manchester	Region: 01-Jun-2015<br>Site, Central: 01-Jun-2015<br>Site, Local: 30-Mar-2016

UNITED KINGDOM	South West – Central Bristol South West REC Centre, Level 3, Block B, Whitefriars, Lewins Mead Bristol UNITED KINGDOM	440802 Level 7 South Block, DD1 9SY, Dundee	Region: 01-Jun-2015 Site, Central: 01-Jun-2015 Site, Local: 29-Jun-2015
UNITED KINGDOM	South West – Central Bristol South West REC Centre, Level 3, Block B, Whitefriars, Lewins Mead Bristol UNITED KINGDOM	440803 Ethelbert Road, Kent, CT1 3NG, Canterbury	Region: 01-Jun-2015 Site, Central: 01-Jun-2015 Site, Local: 23-Jul-2015
UNITED KINGDOM	South West – Central Bristol South West REC Centre, Level 3, Block B, Whitefriars, Lewins Mead Bristol UNITED KINGDOM	440804 Haematology Trial Office Treliske, Truro, TR1 3LJ, Cornwall	Region: 01-Jun-2015 Site, Central: 01-Jun-2015 Site, Local: 23-Jul-2015
UNITED KINGDOM	South West – Central Bristol South West REC Centre, Level 3, Block B, Whitefriars, Lewins Mead Bristol UNITED KINGDOM	440805 West Smithfield, EC1A 7BE, London	Region: 01-Jun-2015 Site, Central: 01-Jun-2015 Site, Local: 27-Aug-2015
UNITED KINGDOM	South West – Central Bristol South West REC Centre, Level 3, Block B, Whitefriars, Lewins Mead Bristol UNITED KINGDOM	440806 Hucknall Road, Nottingham, NG5 1PB, Nottingham	Region: 01-Jun-2015 Site, Central: 01-Jun-2015 Site, Local: 04-Aug-2015
UNITED KINGDOM	South West – Central Bristol South West REC Centre, Level 3, Block B, Whitefriars, Lewins Mead Bristol UNITED KINGDOM	440807 Anchor Unit Foresterhill, AB252ZL, Aberdeen	Region: 01-Jun-2015 Site, Central: 01-Jun-2015 Site, Local: 14-Jul-2015
UNITED KINGDOM	South West – Central Bristol South West REC Centre, Level 3, Block B, Whitefriars, Lewins Mead Bristol UNITED KINGDOM	440808 Wilmslow Road Withington, Manchester, M20 4BX, Manchester	Region: 01-Jun-2015 Site, Central: 01-Jun-2015 Site, Local: 06-Nov-2015
UNITED KINGDOM	South West – Central Bristol South West REC Centre, Level 3, Block B,	440809 St James Institute of Oncology Level 4,	Region: 01-Jun-2015 Site, Central: 01-Jun-2015 Site, Local: 28-Jul-2015

	Whitefriars, Lewins Mead Bristol<br>UNITED KINGDOM	Bexley Wing,<br>Beckett Street, LS9<br>7TF, Leeds	
UNITED<br>KINGDOM	South West – Central Bristol South West<br>REC Centre, Level 3, Block B,<br>Whitefriars, Lewins Mead Bristol<br>UNITED KINGDOM	440810<br>Wednesfield Road,<br>WV10 0QP,<br>Wolverhampton	Region: 01-Jun-2015<br>Site, Central: 01-Jun-2015<br>Site, Local: 26-Nov-2015
UNITED<br>KINGDOM	South West – Central Bristol South West<br>REC Centre, Level 3, Block B,<br>Whitefriars, Lewins Mead Bristol<br>UNITED KINGDOM	440811<br>Derriford Road, PL6<br>8DH, Plymouth	Region: 01-Jun-2015<br>Site, Central: 01-Jun-2015<br>Site, Local: 16-Jul-2015
UNITED<br>KINGDOM	South West – Central Bristol South West<br>REC Centre, Level 3, Block B,<br>Whitefriars, Lewins Mead Bristol<br>UNITED KINGDOM	440812<br>Haematology<br>Department,<br>Mailpoint 8, Level C<br>Tremona Road<br>Hampshire, , SO16<br>6YD, Southampton	Region: 01-Jun-2015<br>Site, Central: 01-Jun-2015<br>Site, Local: 06-Aug-2015
UNITED<br>KINGDOM	South West – Central Bristol South West<br>REC Centre, Level 3, Block B,<br>Whitefriars, Lewins Mead Bristol<br>UNITED KINGDOM	440813<br>Headley Way, OX3<br>7LE, Oxford	Region: 01-Jun-2015<br>Site, Central: 01-Jun-2015<br>Site, Local: 08-Oct-2015
UNITED<br>KINGDOM	South West – Central Bristol South West<br>REC Centre, Level 3, Block B,<br>Whitefriars, Lewins Mead Bristol<br>UNITED KINGDOM	440814<br>250 Euston Road,<br>London	Region: 01-Jun-2015<br>Site, Central: 01-Jun-2015<br>Site, Local: 11-Aug-2015
UNITED<br>STATES	Advarra<br>6100 Merriweather Dr., Suite 600<br>Columbia, MD 21044	100802<br>4725 North Federal<br>Highway Holy Cross<br>Hospital, Florida,<br>33308, Fort<br>Lauderdale	Region: 17-Jun-2015<br>Site, Central: 27-Jan-2015<br>Site, Local: NA

UNITED STATES	Advarra<br>6100 Merriweather Dr., Suite 600<br>Columbia, MD 21044	100803<br>11480 Brookshire Ave Ste 309, California, 90241-5025, Downey	Region: 17-Jun-2015<br>Site, Central: 08-Apr-2015<br>Site, Local: NA
UNITED STATES	Mayo Clinic IRB Office for Human Research Protection 200 First St. SW 201 Building Rm-4-60 Rochester MN 55905	100806<br>200 1st Street Southwest, Minnesota, 55905, Rochester	Region: 17-Jun-2015<br>Site, Central: NA<br>Site, Local: 16-Sep-2015
UNITED STATES	Advarra<br>6100 Merriweather Dr., Suite 600<br>Columbia, MD 21044	100807<br>1021 Morehead Medical Drive 5th Floor, North Carolina, 28204, Charlotte	Region: 17-Jun-2015<br>Site, Central: NA<br>Site, Local: 04-Mar-2015
UNITED STATES	Mayo Clinic IRB Office for Human Research Protection 200 First St. SW 201 Building Rm-4-60 Rochester MN 55905	100809<br>4500 San Pablo Road Florida, 32224, Jacksonville	Region: 17-Jun-2015<br>Site, Central: NA<br>Site, Local: 16-Sep-2015
UNITED STATES	Western Institutional Review Board-Copernicus Group (WCG IRB)<br>212 Carnegie Center, Suite 301<br>Princeton, NJ 08540	100811<br>460 West 10th Avenue<br>Ohio, Columbus 43210	Region: 17-Jun-2015<br>Site, Central: NA<br>Site, Local: 27-Mar-2015
UNITED STATES	Western Institutional Review Board-Copernicus Group (WCG IRB)<br>212 Carnegie Center, Suite 301<br>Princeton, NJ 08540	100812<br>3991 Dutchman's Lane Suite 405, Kentucky, 40207, Louisville	Region: 17-Jun-2015<br>Site, Central: NA<br>Site, Local: 18-Mar-2015
UNITED STATES	BRANY IRB 1981 Marcus Avenue, Suite 210<br>Lake Success, NY 11042	100813<br>One Gustave L Levy Place, New York,	Region: 17-Jun-2015<br>Site, Central: NA<br>Site, Local: 02-Sep-2015

		10029-6574, New York	
UNITED STATES	Western Institutional Review Board-Copernicus Group (WCG IRB)<br>212 Carnegie Center, Suite 301<br>Princeton, NJ 08540	100815<br>2003 Medical Pkwy Suite 210 , Maryland, 21401, Annapolis	Region: 17-Jun-2015<br>Site, Central: NA<br>Site, Local: 30-Mar-2015
UNITED STATES	Advarra<br>6100 Merriweather Dr., Suite 600<br>Columbia, MD 21044	100816<br>220 Page Road, North Carolina, 28374, Pinehurst	Region: 17-Jun-2015<br>Site, Central: 27-Mar-2015<br>Site, Local: NA
UNITED STATES	Advarra<br>6100 Merriweather Dr., Suite 600<br>Columbia, MD 21044	100817<br>3333 Silas Creek Parkway<br>North Carolina, 27103, Winston-Salem	Region: 17-Jun-2015<br>Site, Central: 25-Feb-2015<br>Site, Local: NA
UNITED STATES	The University of Texas Anderson Cancer Center Institutional Review Board U.T. MD Anderson Cancer Center Institutional Review Board<br>7007 Bertner Avenue, Unit 1637<br>Houston, TX 77030-3907	100820<br>1515 Holcombe Blvd., Suite 429 Lymphoma & Myeloma Center, Texas, 77030-4009, Houston	Region: 17-Jun-2015<br>Site, Central: NA<br>Site, Local: 02-Oct-2015
UNITED STATES	Brooke Army Medical Center (BAMC) IRB 3698 Chambers Pass<br>Fort Sam Houston, TX 78234	100821<br>3551 Roger Brooke Dr Attn: Hematology/Oncology , Texas, 78234, Fort Sam Houston	Region: 17-Jun-2015<br>Site, Central: NA<br>Site, Local: 06-Nov-2015
UNITED STATES	Advarra<br>6100 Merriweather Dr., Suite 600<br>Columbia, MD 21044	100823<br>4800 Friendship	Region: 17-Jun-2015<br>Site, Central: 30-Mar-2015<br>Site, Local: NA

		Ave., Pennsylvania, 15224, Pittsburgh	
UNITED STATES	Western Institutional Review Board-Copernicus Group (WCG IRB)<br>212 Carnegie Center, Suite 301<br>Princeton, NJ 08540	100824<br>250 25th Avenue North Suite 412, Tennessee, 37203, Nashville	Region: 17-Jun-2015<br>Site, Central: NA<br>Site, Local: 19-May-2015
UNITED STATES	Sharp Healthcare Institutional Review Board 7930 Frost Street, Suite 300<br>San Diego, CA 92123	100826<br>7901 Frost St, California, 92123, San Diego	Region: 17-Jun-2015<br>Site, Central: NA<br>Site, Local: 30-Mar-2015
UNITED STATES	Western Institutional Review Board-Copernicus Group (WCG IRB) 212 Carnegie Center, Suite 301<br>Princeton, NJ 08540	100827<br>825 Eastlake Ave East Maistop G3-200, Washington, 98109, Seattle	Region: 17-Jun-2015<br>Site, Central: NA<br>Site, Local: 06-Oct-2015
UNITED STATES	Advarra<br>6100 Merriweather Dr., Suite 600<br>Columbia, MD 21044	100828<br>1501 NE Medical Center Dr. Research Dept, Oregon, 97701, Bend	Region: 17-Jun-2015<br>Site, Central: 16-Jan-2015<br>Site, Local: NA
UNITED STATES	Schulman Associates Institutional Review Board 4445 Lake Forest Drive<br>Suite 300<br>Cincinnati OH 45242	100833<br>1211 Coolidge Blvd. Suite 100, Louisiana, 70503, Lafayette	Region: 17-Jun-2015<br>Site, Central: 30-Mar-2015<br>Site, Local: NA
UNITED STATES	Advarra<br>6100 Merriweather Dr., Suite 600<br>Columbia, MD 21044	100837<br>3100 Plaza Properties Blvd., Ohio, 43219, Columbus	Region: 17-Jun-2015<br>Site, Central: 11-Feb-2015<br>Site, Local: NA
UNITED STATES	Advarra<br>6100 Merriweather Dr., Suite 600<br>Columbia, MD 21044	100838<br>5323 South McColl Road, Texas, 78539, Edinburg	Region: 17-Jun-2015<br>Site, Central: 30-Mar-2015<br>Site, Local: NA

UNITED STATES	Advarra<br>6100 Merriweather Dr., Suite 600<br>Columbia, MD 21044	100839<br>7910 W. Jefferson Blvd., Suite 108, Indiana, 46804, Fort Wayne	Region: 17-Jun-2015<br>Site, Central: 30-Mar-2015<br>Site, Local: NA
UNITED STATES	Avera Institutional Review Board 5300 S. Broadband Lane Sioux Falls, South Dakota 57108 UNITED STATES	100841<br>Prairie Center 1000 East 23rd Street, Suite 230, South Dakota, 57105-1018, Sioux Falls	Region: 17-Jun-2015<br>Site, Central: NA<br>Site, Local: 24-Jun-2015
UNITED STATES	Schulman Associates Institutional Review Board 4445 Lake Forest Drive<br>Suite 300<br>Cincinnati OH 45242	100843<br>4875 Higbee Ave NW, Ohio, 44718, Canton	Region: 17-Jun-2015<br>Site, Central: 05-Mar-2015<br>Site, Local: NA
UNITED STATES	Henry Ford Health System Institutional Review Board 1 Ford Place<br>Research Administration, 2F<br>Detroit, MI USA 48202-3450	100853<br>2799 West Grand Blvd. K-13, Detroit Michigan 48202	Region: 17-Jun-2015<br>Site, Central: NA<br>Site, Local: 11-Jun-2015
UNITED STATES	Human Research Protections Office (HRPO), University of Maryland, Baltimore 620 W. Lexington Street, Second Floor<br>Baltimore, Maryland 21201	100854<br>Greenebaum Cancer Center 22 South Greene Street, Maryland, 21201, Baltimore	Region: 17-Jun-2015<br>Site, Central: NA<br>Site, Local: 07-Oct-2015
UNITED STATES	IntegReview IRB<br>3815 S. Capital of Texas Hwy<br>Suite 320 Austin, TX, USA, 78704	100855<br>522 Timberdale Lane Texas, 77090, Houston	Region: 17-Jun-2015<br>Site, Central: NA<br>Site, Local: 07-Aug-2015
UNITED STATES	University of Coloradoo Health Institutional Review Board 3702 Automation Way, Suite 200 Fort Collins, CO 80525 UNITED STATES	100856<br>2121 E. Harmony Road Suite 170,	Region: 17-Jun-2015<br>Site, Central: NA<br>Site, Local: 04-Jun-2015

		Colorado, 80528, Fort Collins	
UNITED STATES	Columbia University Medical Center IRB 154 Haven Avenue, Floor 1 New York, NY 10032 UNITED STATES	100857<br>161 Fort Washington Ave, New York, 10032, New York	Region: 17-Jun-2015<br>Site, Central: NA<br>Site, Local: 19-Jan-2016
UNITED STATES	Western Institutional Review Board-Copernicus Group (WCG IRB) 212 Carnegie Center, Suite 301<br>Princeton, NJ 08540	100859<br>2205 McCallie Avenue Suite 1000, Tennessee, 37404, Chattanooga	Region: 17-Jun-2015<br>Site, Central: NA<br>Site, Local: 28-May-2015
UNITED STATES	Schulman Associates Institutional Review Board 4445 Lake Forest Drive<br>Suite 300<br>Cincinnati OH 45242	100860<br>8900 Wilshire Boulevard, California, 90211, Beverly Hills	Region: 17-Jun-2015<br>Site, Central: 19-May-2015<br>Site, Local: NA
UNITED STATES	Dana-Farber Cancer Institute IRB 450 Brookline Avenue, OS-229<br>Boston, MA 02115	100862<br>55 Fruit Street #531, Massachusetts, 2114, Boston	Region: 17-Jun-2015<br>Site, Central: NA<br>Site, Local: 11-Aug-2015
UNITED STATES	Advarra<br>6100 Merriweather Dr., Suite 600<br>Columbia, MD 21044	100868<br>101 E. Wood St, South Carolina, 29303, Spartanburg	Region: 17-Jun-2015<br>Site, Central: 14-Aug-2015<br>Site, Local: NA
UNITED STATES	Advarra<br>6100 Merriweather Dr., Suite 600<br>Columbia, MD 21044	100870<br>2316 E Meyer Blvd, Missouri, 64132, Kansas City	Region: 17-Jun-2015<br>Site, Central: 29-May-2015<br>Site, Local: NA
UNITED STATES	Texas Health Resources Institutional Review Board 612 E. Lamar Blvd, Suite 1212, Arlington, TX 76011	100872<br>6957 West Plano Parkway #2000A, Texas, 75093, Plano	Region: 17-Jun-2015<br>Site, Central: NA<br>Site, Local: 12-May-2015

UNITED STATES	Advarra<br>6100 Merriweather Dr., Suite 600<br>Columbia, MD 21044	100874<br>5290 South 400 East<br>Utah, 84405, Ogden	Region: 17-Jun-2015<br>Site, Central: 29-Apr-2015<br>Site, Local: NA
UNITED STATES	Advarra<br>6100 Merriweather Dr., Suite 600<br>Columbia, MD 21044	100875<br>2130 W. Holcombe Blvd 10th floor,<br>77030, Houston	Region: 17-Jun-2015<br>Site, Central: 28-May-2015<br>Site, Local: NA
UNITED STATES	Western Institutional Review Board-Copernicus Group (WCG IRB)<br>212 Carnegie Center, Suite 301<br>Princeton, NJ 08540	100876<br>Valley View Hospital<br>1906 Blake Ave,<br>Colorado, 81601,<br>Glenwood Springs	Region: 17-Jun-2015<br>Site, Central: NA<br>Site, Local: 21-Jul-2015
UNITED STATES	Advarra<br>6100 Merriweather Dr., Suite 600<br>Columbia, MD 21044	100877<br>6001 North Mayfair Street, Washington,<br>99208-1129, Spokane	Region: 17-Jun-2015<br>Site, Central: 23-Apr-2015<br>Site, Local: NA
UNITED STATES	Advarra<br>6100 Merriweather Dr., Suite 600<br>Columbia, MD 21044	100886<br>1062 Forsyth Street,<br>Georgia, 31201,<br>Macon	Region: 17-Jun-2015<br>Site, Central: 19-Jul-2016<br>Site, Local: NA
UNITED STATES	Advarra<br>6100 Merriweather Dr., Suite 600<br>Columbia, MD 21044	100888<br>3617 Vista Way,<br>California, 92056,<br>Oceanside	Region: 17-Jun-2015<br>Site, Central: 05-May-2015<br>Site, Local: NA
UNITED STATES	Advarra<br>6100 Merriweather Dr., Suite 600<br>Columbia, MD 21044	100890<br>373 North Fayetteville Street,<br>North Carolina,<br>27203, Asheboro	Region: 17-Jun-2015<br>Site, Central: 26-May-2015<br>Site, Local: NA
UNITED STATES	Western Institutional Review Board-Copernicus Group (WCG IRB)<br>212 Carnegie Center, Suite 301<br>Princeton, NJ 08540	100892<br>601 Elmwood Ave<br>#704 James P.<br>Wilmot Cancer	Region: 17-Jun-2015<br>Site, Central: NA<br>Site, Local: 11-Sep-2015

		Center, New York,<br>14642-0001,<br>Rochester	
UNITED STATES	Cleveland Clinic Institutional Review Board<br>9550 Euclid Avenue OS-1<br>Cleveland, OH USA 44195	100895<br>Cleveland Clinic Florida – Oncology<br>2950 Cleveland Clinic Boulevard.<br>Fort Lauderdale, FL, 33331	Region: 17-Jun-2015<br>Site, Central: NA<br>Site, Local: 13-May-2016
UNITED STATES	St Lukes Hosptial IRB 915 Eat First Street, Duluth, MN 55805	100896<br>915 East 1st st, Minnesota, 55805, Duluth	Region: 17-Jun-2015<br>Site, Central: NA<br>Site, Local: 14-Sep-2015
UNITED STATES	FMH Institutional Revew Board 400 West Seventh Street, Frederick, MD 21701	100897<br>1562 Opossumtown Pike Maryland, 21701, Frederick	Region: 17-Jun-2015<br>Site, Central: NA<br>Site, Local: 15-Jun-2015
UNITED STATES	INSTITUTIONAL REVIEW BOARD<br>770~~23~~ Floyd Curl Drive, Mail Code 7830<br>San Antonio TX, 78229	100899<br>7979 Wurzbach Road, Zeller Bldg, 4th floor, MC8232, 78229, San Antonio	Region: 17-Jun-2015<br>Site, Central: NA<br>Site, Local: 17-Jul-2015
UNITED STATES	Western Institutional Review Board-Copernicus Group (WCG IRB)<br>212 Carnegie Center, Suite 301<br>Princeton, NJ 08540	100903<br>1201 5th Avenue North Suite 505, Florida, 33705, St. Petersburg	Region: 17-Jun-2015<br>Site, Central: NA<br>Site, Local: 17-Jul-2015
UNITED STATES	Western Institutional Review Board-Copernicus Group (WCG IRB)<br>212 Carnegie Center, Suite 301<br>Princeton, NJ 08540	100904<br>4331 Veronica S. Shoemaker Unit 15,	Region: 17-Jun-2015<br>Site, Central: NA<br>Site, Local: 17-Jul-2015

		Florida, 33916, Fort Myers	
UNITED STATES	Cleveland Clinic Institutional Review Board (IRB Cleveland Clinic Foundation9500 Euclid Avenue Cleveland, OH 44195	100907<br>9500 Euclid Avenue HB-105, Ohio, 44195, Cleveland	Region: 17-Jun-2015<br>Site, Central: NA<br>Site, Local: 21-Sep-2015
UNITED STATES	Advarra<br>6100 Merriweather Dr., Suite 600<br>Columbia, MD 21044	100909<br>701 NW 13th Street Room 3013, Florida, 33486, Boca Raton	Region: 17-Jun-2015<br>Site, Central: 16-Feb-2016<br>Site, Local: NA
UNITED STATES	Western Institutional Review Board<br>1019 39th Avenue SE, Suite 120<br>Puyallup, Washington 98374-2115	100910<br>Emory University - Winship C.I.<br>1365-C Clifton Road NE<br>Atlanta, Georgia 30322	Region: 17-Jun-2015<br>Site, Central: NA<br>Site, Local: 18-Mar-2016
UNITED STATES	University of Nebraska Medical Center IRB University of Nebraska Medical Center, 42nd and Emile, Omaha, NE 68198	100911<br>987680 Nebraska Medical Center Nebraska, 68198-7680, Omaha	Region: 17-Jun-2015<br>Site, Central: NA<br>Site, Local: 18-Mar-2016
UNITED STATES	RWJS IRB 110 Rehill Ave, Somerville, NJ 08876	100914<br>30 Rehil Avenue Suite 2500, New Jersey, 8876, Somerville	Region: 17-Jun-2015<br>Site, Central: NA<br>Site, Local: 25-Nov-2015
UNITED STATES	BRANY IRB 1981 Marcus Avenue, Suite 210<br>Lake Success, NY 11042	100916<br>20 York St, Connecticut, 06510-3220, New Haven	Region: 17-Jun-2015<br>Site, Central: NA<br>Site, Local: 18-Nov-2015

UNITED STATES	Dana-Farber Cancer Institute IRB 450 Brookline Avenue, OS-229 Boston, MA 02115	100917 450 Brookline Avenue, Massachusetts, 02215, Boston	Region: 17-Jun-2015<br>Site, Central: NA<br>Site, Local: 11-Aug-2015

Supplementary Table 2. Demographic and baseline disease characteristics in the ITT population

	D-Rd (n=368)	Rd (n=369)
Median age, years	73 (70–78)	74 (70–78)
Age category		
<65 years	4 (1%)	4 (1%)
65 to <70 years	74 (20%)	73 (20%)
70 to <75 years	130 (35%)	131 (36%)
≥75 years	160 (43%)	161 (44%)
≥80 years	66 (18%)	71 (20%)
Sex[a]		
Male	189 (51%)	195 (53%)
Female	179 (49%)	174 (47%)
Race[a]		
White	336 (91%)	339 (92%)
Black or African American	12 (3%)	16 (4%)
Asian	3 (1%)	2 (1%)
Native Hawaiian or other Pacific Islander	0	1 (<1%)
Other[b]	6 (2%)	6 (2%)
Unknown/not reported	11 (3%)	5 (1%)
Ethnicity		
Hispanic or Latino	11 (3%)	12 (3%)
Not Hispanic or Latino	347 (94%)	352 (95%)
Unknown/not reported	10 (3%)	5 (1%)
ECOG PS score[c]		
0	127 (35%)	123 (33%)
1	178 (48%)	187 (51%)
≥2[d]	63 (17%)	59 (16%)
ISS disease stage[e]		
I	98 (27%)	103 (28%)
II	163 (44%)	156 (42%)
III	107 (29%)	110 (30%)
Type of measurable disease		
IgG	225 (61%)	231 (63%)
IgA	65 (18%)	66 (18%)
Other[f]	9 (2%)	10 (3%)
Detected in urine only	40 (11%)	34 (9%)
Detected as serum FLC only	29 (8%)	28 (8%)
Cytogenetic risk profile[g]		
Standard risk	271/319 (85%)	279/323 (86%)
High risk	48/319 (15%)	44/323 (14%)
Median time since initial diagnosis of multiple myeloma, months	0.95 (0.53–1.46)	0.89 (0.59–1.45)

Data are median (interquartile range), n (%), or n/N (%). The ITT population included all patients who underwent randomization.

ITT, intention-to-treat; D-Rd, daratumumab plus lenalidomide/dexamethasone; Rd, lenalidomide/dexamethasone; ECOG PS, Eastern Cooperative Oncology Group performance status; ISS, International Staging System; FLC, free light chain.

[a]Sex and race were self-reported by patients.

[b]Patients reporting multiple races.

[c]ECOG PS is scored on a scale of 0 to 5, with 0 indicating no symptoms and higher scores indicating increasing disability.

[d]Two patients had a score of >2 (one patient had a score of 3, and another patient had a score of 4).

[e]ISS disease stage was based on the combination of serum β2-microglobulin and albumin.

[f]This category includes IgD, IgE, IgM, and biclonal gammopathies.

[g]Cytogenetic risk was based on fluorescence in situ hybridization or karyotype analysis; patients who had a high-risk cytogenic profile had ≥1 of the following high-risk abnormalities: del(17p), t(14;16), or t(4;14).

Supplementary Table 3. Summary of patients who did not receive subsequent antimyeloma therapy in the ITT population

	D-Rd (n=368)	Rd (n=369)
Total number of patients who did not receive any subsequent antimyeloma therapy during the study	228 (62%)	168 (46%)
Patients who discontinued the study[a]	228 (100%)	168 (100%)
Reason for discontinuation[a]		
Death while on study treatment[b]	30 (13%)	34 (20%)
Physician decision	1 (<1%)	0
Patient withdrawal or lost to follow-up	14 (6%)	23 (14%)
Other reasons[a,c]	183 (80%)	111 (66%)
Death after study treatment	46 (20%)	42 (25%)
Other – end of data collection	137 (60%)	68 (40%)
Patients who were still on study treatment at study end	79 (35%)	20 (12%)
Other[d]	0	1 (1%)

Data are n (%).
ITT, intention-to-treat; D-Rd, daratumumab plus lenalidomide/dexamethasone; Rd, lenalidomide/dexamethasone.
[a]Percentages are based on the number of patients in the ITT population who did not receive any subsequent antimyeloma therapy.
[b]Death while on study treatment was defined as study discontinuation due to death, with the death occurring within 30 days after the last study treatment. Death after study treatment was defined as study discontinuation due to death, with the death occurring >30 days after the last study treatment.
[c]Other reasons include death and other.
[d]Other includes "end of data collection".

Supplementary Figure 1. OS by MRD (10^{-5}) status and treatment group in the ITT population.

MRD positive consists of ITT patients who were not tested and assumed not negative or tested and found positive or indeterminate.

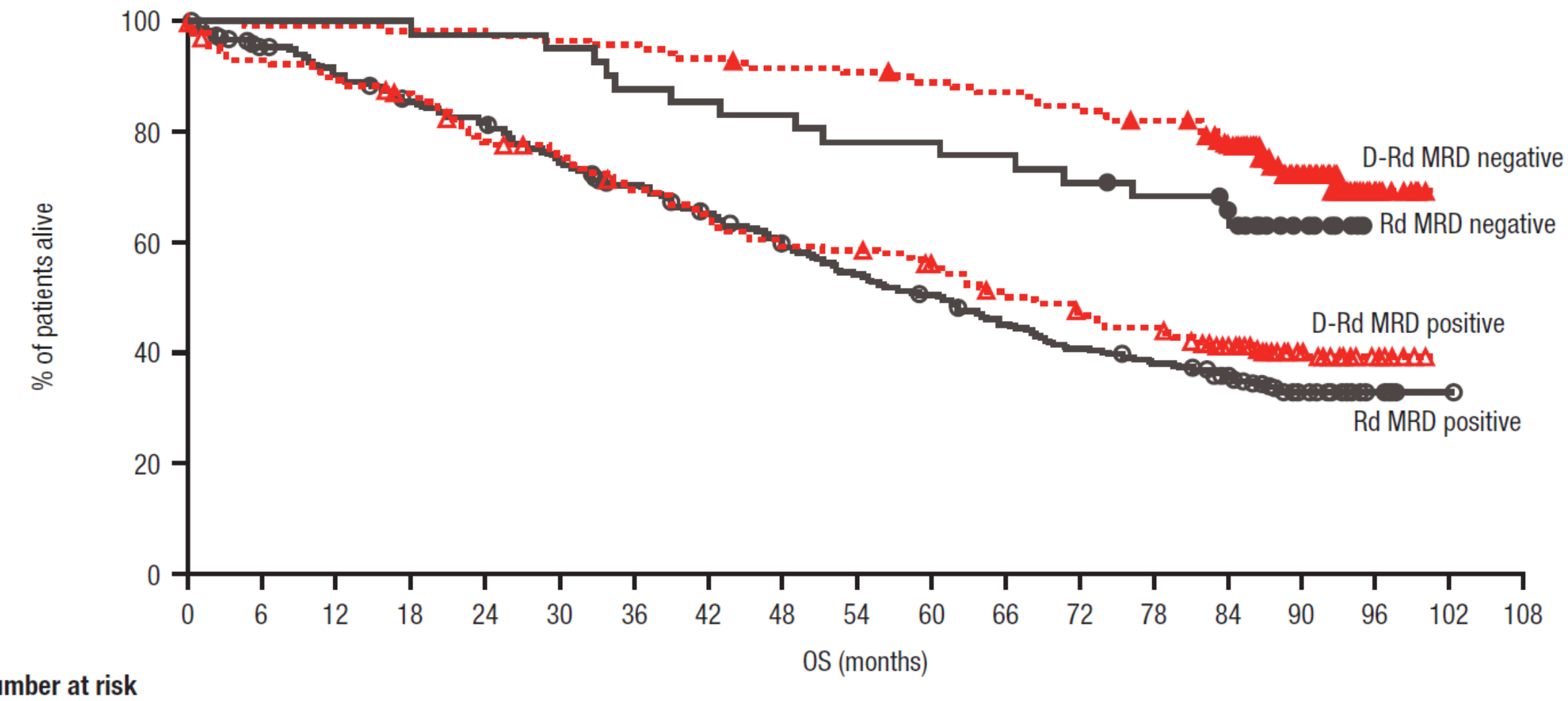

Number at risk (number censored)	0	6	12	18	24	30	36	42	48	54	60	66	72	78	84	90	96	102	108
D-Rd MRD negative	118 (0)	117 (0)	117 (0)	116 (0)	115 (0)	114 (0)	113 (0)	110 (0)	107 (1)	106 (1)	102 (3)	100 (3)	97 (3)	93 (4)	81 (11)	40 (48)	7 (80)	0 (87)	0 (87)
Rd MRD negative	41 (0)	41 (0)	41 (0)	41 (0)	40 (0)	39 (0)	36 (0)	35 (0)	34 (0)	32 (0)	32 (0)	31 (0)	29 (0)	27 (1)	24 (3)	10 (16)	0 (26)	0 (26)	0 (26)
D-Rd MRD positive	250 (0)	229 (3)	221 (3)	212 (5)	190 (6)	183 (8)	167 (9)	156 (9)	142 (9)	140 (9)	131 (12)	117 (13)	109 (14)	102 (14)	87 (22)	50 (57)	14 (92)	0 (106)	0 (106)
Rd MRD positive	328 (0)	302 (13)	283 (14)	267 (16)	254 (16)	231 (17)	215 (20)	197 (22)	179 (24)	162 (24)	150 (25)	133 (26)	120 (26)	111 (27)	96 (36)	49 (76)	11 (114)	2 (123)	0 (125)

OS, overall survival; MRD, minimal residual disease; ITT, intention-to-treat;
D-Rd, daratumumab plus lenalidomide/dexamethasone; Rd, lenalidomide/dexamethasone.

Supplementary Figure 2. OS by sustained MRD negativity (10^{-5}) status and treatment group in the ITT population.

Sustained MRD negativity is defined as MRD negative and confirmed by ≥1 year apart without MRD positive in between.

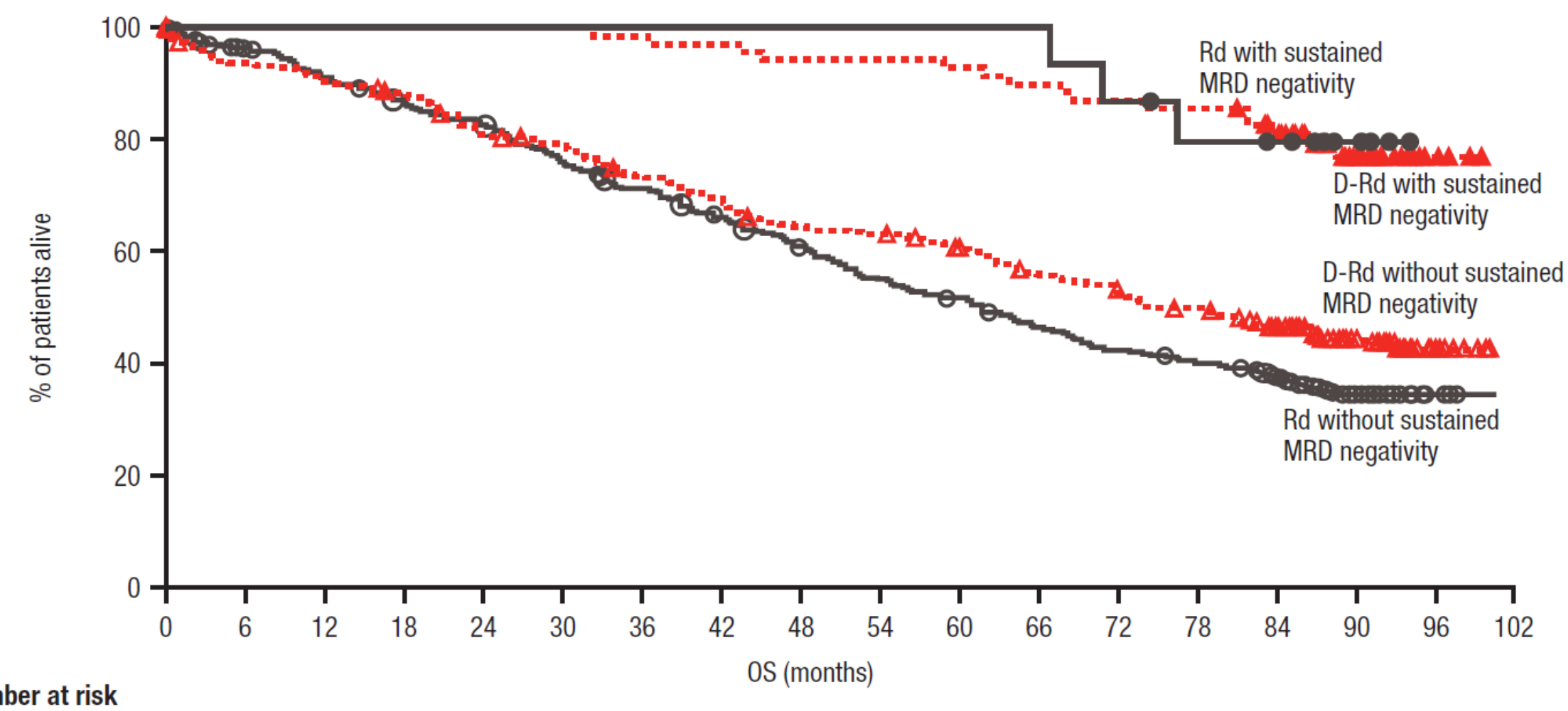

Number at risk (number censored)

	0	6	12	18	24	30	36	42	48	54	60	66	72	78	84	90	96	102
D-Rd with sustained MRD negativity	69 (0)	69 (0)	69 (0)	69 (0)	69 (0)	69 (0)	68 (0)	67 (0)	65 (0)	65 (0)	64 (0)	62 (0)	60 (0)	59 (0)	51 (5)	27 (27)	5 (49)	0 (54)
Rd with sustained MRD negativity	15 (0)	15 (0)	15 (0)	15 (0)	15 (0)	15 (0)	15 (0)	15 (0)	15 (0)	15 (0)	15 (0)	15 (0)	13 (0)	11 (1)	10 (2)	5 (7)	0 (12)	0 (12)
D-Rd without sustained MRD negativity	299 (0)	277 (3)	269 (3)	259 (5)	236 (6)	228 (8)	212 (9)	199 (9)	184 (10)	181 (10)	169 (15)	155 (16)	146 (17)	136 (18)	117 (28)	63 (78)	16 (123)	0 (139)
Rd without sustained MRD negativity	354 (0)	328 (13)	309 (14)	293 (16)	279 (16)	255 (17)	236 (20)	217 (22)	198 (24)	179 (24)	167 (25)	149 (26)	136 (26)	127 (27)	110 (37)	54 (85)	11 (128)	2 (137)

OS, overall survival; MRD, minimal residual disease; ITT, intention-to-treat;
D-Rd, daratumumab plus lenalidomide/dexamethasone; Rd, lenalidomide/dexamethasone.

Supplementary Figure 3. Best response to first subsequent antimyeloma therapy in the ITT population.

Data represent the best response to the first subsequent systemic antimyeloma therapy recorded for patients who went on to receive subsequent therapy and were evaluable for their best response to their first subsequent therapy. Percentages were calculated with the number of patients in each group who received subsequent therapy and had available data on their best response to their first subsequent therapy as the denominator (D-Rd, n=130; Rd, n=193). Total percentage may exceed 100% due to rounding.

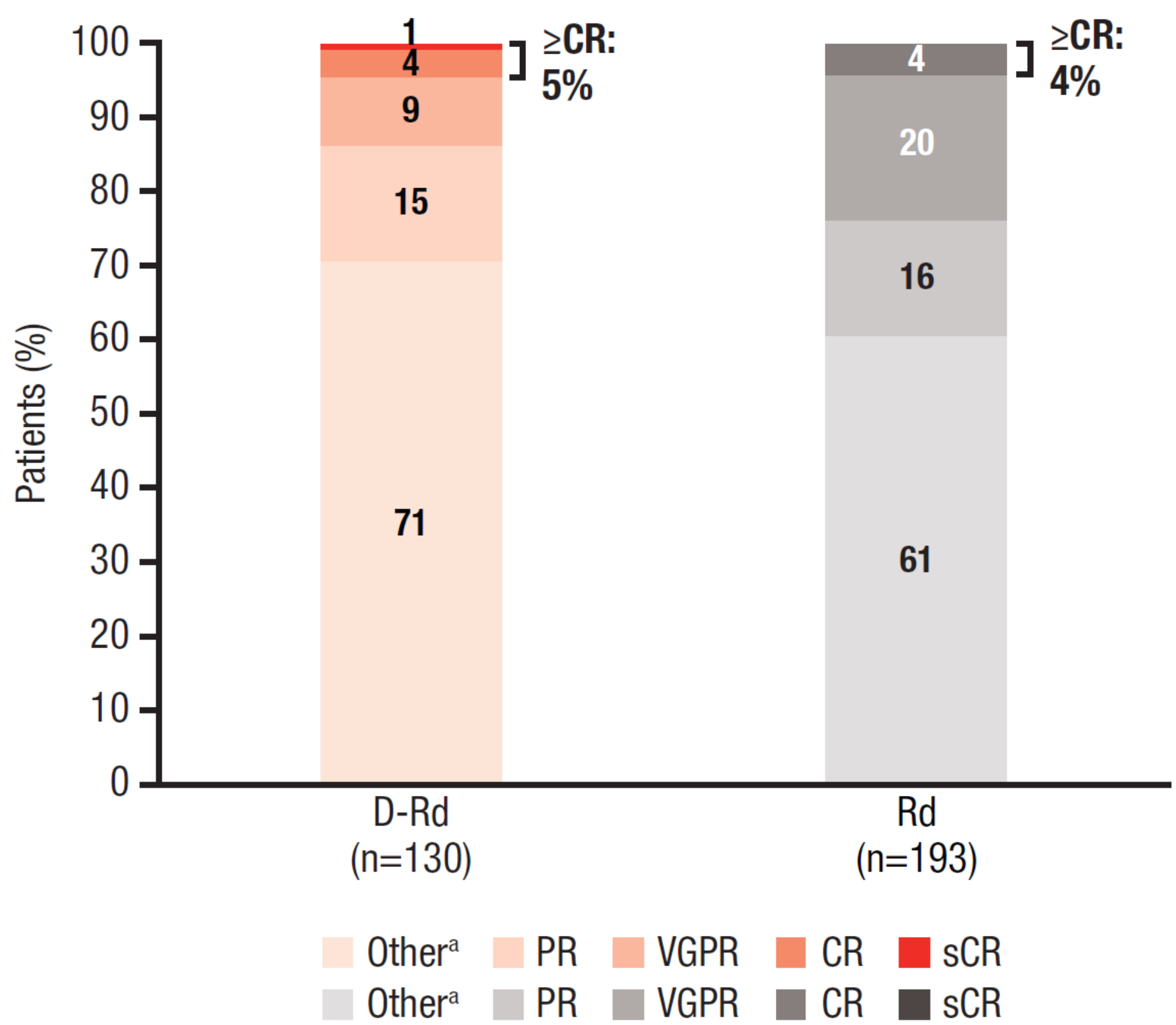

ITT, intention-to-treat; CR, complete response; D-Rd, daratumumab plus lenalidomide/dexamethasone; Rd, lenalidomide/dexamethasone; PR, partial response; VGPR< very good partial response; sCR, stringent complete response.

[a]Other includes the following response categories: minimal response, stable disease, progressive disease, not applicable, and unknown.

References

1. Rajkumar SV, Harousseau JL, Durie B, Anderson KC, Dimopoulos M, Kyle R, et al. Consensus recommendations for the uniform reporting of clinical trials: report of the International Myeloma Workshop Consensus Panel 1. Blood. 2011;117:4691–4695.
2. Durie BGM, Harousseau JL, Miguel JS, Bladé J, Barlogie B, Anderson K, et al. International uniform response criteria for multiple myeloma. Leukemia. 2006;20:1467–1473.
